# *Broussonetia papyrifera* Pollen Metabolome Insights, Allergenicity, and Dispersal in Response to Climate Change Variables

**DOI:** 10.3390/metabo15020137

**Published:** 2025-02-18

**Authors:** Muhammad Humayun, Saadia Naseem, Richard E. Goodman, Zahid Ali

**Affiliations:** 1 Plant Biotechnology and Molecular Pharming Lab, Department of Biosciences, COMSATS University Islamabad (CUI), Islamabad 45550, Pakistan; humayun515@gmail.com (M.H.); saadia.naseem@comsats.edu.pk (S.N.); 2 Food Allergen Research and Resource Program, Department of Food Science and Technology, University of Nebraska-Lincoln, Lincoln, NE 68588, USA; rgoodman2@unl.edu

**Keywords:** *B. papyrifera* pollen, FTIR, LC-MS/MS, climate change, pollen allergy

## Abstract

**Background/Objectives**: *Broussonetia papyrifera* is a tree-producing allergenic pollen that grows in varied climatic conditions worldwide and causes pollen allergies in susceptible humans. This study aimed to investigate *B. papyrifera* pollen morphology, pollen metabolome, pollen allergenicity, and climate change’s impact on the plant habitat suitability in the future. **Methods**: Tree pollen was collected in spring from different regions of Pakistan. Pollen samples were subjected to morphological analysis, Fourier transform infrared spectroscopy (FTIR), liquid chromatography–mass spectrometry (LC-MS/MS), and immunoblotting. **Results**: MaxEnt modeling predicted the tree’s future-growth invasion into new regions. Scanning electron microscopy (SEM) and FTIR displayed regional differences in pollen morphology and metabolome correlated to shifts in climatic variables. LC-MS/MS analysis detected four lipids that can potentially stimulate inflammatory responses. Pollen protein immunoblotting studies identified a putative 15 kDa novel allergen and verified previously known 40 kDa, 33 kDa, and 10 kDa allergens. *B. papyrifera* MaxEnt modeling through ACCESS1.0 and CCSM4 under 2-greenhouse gas emissions scenarios {representative concentration pathway (RCP) 4.5 and 8.5} projected the tree invasion by the years 2050 and 2070. **Conclusions**: The study findings demonstrate that differences in climatic variables affect *B. papyrifera*-pollen metabolome and predict the habitat suitability of the tree for invasion in the future. The study results provide a model system for studying other species’ pollen morphology, metabolome, future habitat suitability for plant invasion, and associated allergies in response to climate change.

## 1. Introduction

Climate change is expected to have significant effects on plant growth and development and could impact pollen seasons [1]. The climate change conditions have affected plants [2]. Pollen, small in size, remains in the air for longer periods and is transported to longer distances [3,4]. Pollen has been widely studied in forensics, ecology, climate change, and aeroallergens investigations [5]. Aeroallergen research suggests an increase in the concentration of some allergenic pollen taxa and pollen allergies over the past 50 years [6]. This may be due to shifts in meteorological variables and an increase in air pollutants, which changed pollen quantity in the air in distinct bioclimatic regions [7]. An increase in temperature influences carbohydrates, proteins, and lipids levels in several pollen taxa [8]. Higher temperatures and elevated carbon and nitrogen concentrations affect pollen development [9]. Developing pollen requires a substantial amount of membrane lipids and fatty acids [10,11]. Temperature stress during pollen development affects pollen tube growth [12], pollen appearance, wall, and vesicular transport [13]. Increasing temperature causes an early bursting of the pollen tapetum layer, which may alter its allergenicity. The pollen is rich in mitochondria [14], and oxidative damage in the tapetum layer leads to increased production of reactive oxygen species (ROS), which causes programmed cell death [15]. ROS activation causes the regulation of pollen-associated lipid metabolites (PALMs) and phytoprostanes, which have been shown to evoke immune responses [16,17]. Oxylipins are involved in plants’ growth and development and defend them from abiotic stresses [16]. Similarly, increasing ozone levels due to climate change have enhanced Birch pollen allergenic potential [18].

*Broussonetia papyrifera* is anemophilous (wind-pollinated), and its pollen is allergenic [19,20,21]. The pollen protein extracts analysis by skin prick test (SPT) and enzyme-linked immune sorbent assay (ELISA) identified 33-kDa and 40-kDa proteins as putative allergens that may elicit clinical responses in humans. The tree pollen is reported to cause urticaria and severe respiratory symptoms [22]. Sequence identities of the pollen IgE binding proteins have not been identified, and the tree produces large quantities of pollen of 12–17 µm size [23]. In Islamabad, Pakistan, *B. papyrifera*’s daily pollen count records show the highest concentration of 40,000 pollen grains/m^3^ in the spring season [24]. According to the Global Diversity Information Facility (GBIF), *B. papyrifera* grows in Asia, in tropical and subtropical regions near the equator. This species was introduced in Pakistan in the early 1960s from East Asia for the beautification of Islamabad [20]. There is remarkable variation in climatic factors in different regions of Pakistan (average temperature and precipitation) [25,26].

Pollen exine morphology can be studied through microscopic techniques [27]. However, studying changes in pollen lipids, proteins, and carbohydrate constituents requires molecular approaches. Variations in the biochemical composition of pollen through Fourier transform infrared spectroscopy (FTIR) are informative for understanding the complex changes that can occur [28]. Kendel and Zimmermann have shown relative and absolute contents of lipids, carbohydrates, carotenoids, proteins, and sporopollenins. FTIR has been used as an easy and quick approach to study the biochemical composition of pollen where environmental stresses affect natural pollen samples [29,30]. Similarly, pollutants can affect pollen and intensify pollen allergies in humans [31]. Liquid chromatography–mass spectrometry (LC-MS/MS) can measure the presence of various chemical compounds in a sample by combining chromatography and mass spectrometry [32]. This study aims to find inter-regional variations in *B. papyrifera* pollen in varied climatic conditions, examine *B. papyrifera* pollen allergenicity, and predict the tree’s future habitat suitability.

## 2. Materials and Methods

### 2.1. Study Area Characteristics

The geographical and climatic conditions of the study area are different, including district Peshawar Region 1 (R1), district Islamabad Region 2 (R2), and district Kotli Region 3 (R3). R1 is located at 331 m, R2 at 507 m, and R3 at 609 m elevation from sea level. The distance between R1 and R3 is 316 km; R1 and R2 is 187 km; and R2 and R3 is 132 km. The mean monthly temperature during spring for Region 1, Region 2, and Region 3 was 22.9 °C, 21.9 °C and 22.1 °C, respectively, and mean monthly precipitation during the period was 5.2 mm for R1, 59.5 mm for R2, and 21.1 mm for R3 [33]. *B. papyrifera* is allergenic and commonly grows in the three climatic regions. Regions R2 and R3 have lower average annual temperatures and increased mean annual precipitation than R1, which has increased mean annual temperature and decreased mean annual precipitation. All study regions have variations in bioclimatic variables (Table S1, Figures S1 and S2).

### 2.2. B. papyrifera Growth Occurrence Record

The distribution of *B. papyrifera* in Asia was downloaded from the GBIF. Additionally, a field observation survey was carried out in *B. papyrifera* growing regions in Pakistan in 2020 and 2021 to record the plant occurrence. In the *B. papyrifera* growing regions survey, a minimum of 1 km of distant locations’ longitudes and latitudes were recorded. However, the nearest accessible location was recorded for plants growing in the mountainous region, where recording the exact location was difficult. The presence of the tree was recorded through the Google Earth Pro version 7.3 mobile application. The field observation data combined with GBIF.org (accessed on 11 April 2022) data showed a record of 394 points in a dataset (See Table S2). The refined data were entered in a Microsoft Excel sheet for species with data (SWD) in CSV format to build the MaxEnt model (see Table S2).

### 2.3. Pollen Sampling

Mature pollen samples were collected from three *B. papyrifera* trees in each region growing at random spots in R1, R2, and R3 (Table S3, Figures S3 and S4) in the spring of 2020 and 2021. All sites were classified as green parks in urban habitats away from roads. Sterilized 50 mL tubes were used to store flowers to avoid pollen contamination from external sources. The samples were dried for 72 h in the shade at room temperature. Dried pollen grains were released from the samples by tipping the dry panicle of *B. papyrifera* on a 500 µm pore size mesh. The isolated pollen grains were then passed through a 100 µm pore size mesh and a 50 µm pore size mesh to collect pure pollen grains. The pure pollen samples were stored at room temperature (28 °C) in 1.5 mL sterile tubes.

### 2.4. Scanning Electron Microscopy of Pollen Samples

Scanning electron microscopy (SEM) was performed on dried *B. papyrifera* pollen samples on HITACHI SU 1500 equipment. Pollen was directly kept on SEM stub holders covered by a carbon patch. Pollen images were taken at 5 KV accelerating voltage in a high vacuum mode using SE detector at 50 µm resolution, and pollen diameters were calculated using the microscale tool of the SEM [34].

### 2.5. Fourier Transform Infrared Analysis of Pollen

Samples of 20 mg of dried pollen grains taken from each sample of *B. papyrifera* were placed on FTIR IRTracer-100 knob for analysis following the protocol as described by Kendel and Zimmerman [28]. Measurements were taken in triplicate with a Vertex 70 v FTIR Spectrometer using the attenuated total reflectance (ATR) method. Infrared radiations in the range of 500–4500 cm^−1^ and a resolution of 4 cm^−1^ were selected to draw peaks [35]. The peaks were normalized, and data files were imported in CSV format, which was processed to draw FTIR peaks through Origin Pro 8.5E-2018 software.

### 2.6. Liquid Chromatography–Mass Spectrometry Analysis of Pollen

Samples of dried pollen (100 mg) were placed in 15 mL tubes with 2 mL of methanol as an extraction fluid, and the tubes were kept in a shaker at room temperature for 1 h. The extracts were passed through a Solid Phase Extraction (SPE) assembly, and samples were collected in 2.0 mL tubes. Pollen extracts were subjected to LC-MS/MS analysis [36]. An Agilent triple quadrupole system was used for LC-MS/MS where compounds were separated (model: G1776A; dimensions: 160 mm × 435 mm × 436 mm; temperature: 40 °C). The injection volume of the sample was 5 μL. The mobile phase contained solvent A (100% acetonitrile) and solvent B (100% water). The mobile phase flow rate was 0.3 mL min^−1^. The HPLC gradient included 0–4 min. 2% A, 98% B; 4–7 min. 20% A, 80% B; 7–14 min. 90% A, 10% B; 15 min. 90% A, 10% B; 17 min. 2% A and 98% B. The ESI positive mode using nitrogen gas was used for mass spectrometry at 350 °C, with an 11 L min^−1^ gas flow, 4500 v capillary voltage, and r 50 psi nebulizer pressure to determine compounds. Identified compounds were compared with the National Institute of Standards and Technology (NIST)-17 database.

### 2.7. Pollen Protein Extracts Separated in SDS-PAGE Followed by Immunoblotting

Water-soluble pollen proteins were extracted in Bio-Rad^TM^ 1X PBS buffer Cat # 1610763. Extracted proteins were quantified by Thermo Scientific NanoDrop 2000C and using a Cytiva^TM^ 2D-Quant kit Cat # 80648354. Samples of 10 µg protein extract/well were separated in Novex 10–20% tris-glycine gels (Invitrogen^TM^, Carlsbad, CA, USA, Cat # EC61355). A 3 μL sample of pre-stained Precision Plus molecular weight marker proteins was loaded (Bio-Rad, Hercules, CA, USA, Cat # 161-0374) on each gel to allow proteins apparent molecular weight estimation. Protein extract IgE binding performed using individual serum samples was confirmed by native immune-dot blot IgE binding activity of protein extracts with monoclonal mouse anti-human IgE conjugated with horseradish peroxidase (HRP) from Southern Biotech, Birmingham, AL (clone B3102E8 Cat # 9160-05). Human serum samples were purchased from Plasma Lab USA (Table S5). Allergens were confirmed through Western blotting and immune-dot blots with selected serum samples. Protein extracts were pre-heated at 25 °C, 70 °C, and 95 °C, cooled to room temperature, and then separated into premade gels (Novex). Proteins were transferred to 0.45 µm nitrocellulose membranes (Invitrogen^TM^, Carlsbad, CA, USA, Cat # 645,239) and blocked with non-fat milk solution before applying diluted serum samples. Unbound IgE was removed by washing with 0.2% Tween 20 in PBS and incubated with Southern Biotech’s monoclonal anti-IgE combined with horse radish peroxidase. The anti-IgE was visualized using Supersignal^TM^ West Dura Extended Duration Chemiluminescent substrate (Pierce, Rockford, IL, USA, Cat # 34,076) in a UVP BioSpectrum 815 Imaging System for 1 min and 5 min as described by [37].

### 2.8. Temperature, Precipitation, and Spring Maximum Pollen Count Data Analysis

Mean monthly temperature and precipitation data from 2010 to 2020 for districts R1, R2, and R3 were retrieved from the Meteorological Department (PMD). Single day *B. papyrifera* maximum pollen concentrations per cubic meter during spring in R2 were retrieved from the PMD. GraphPad Prism 2.0 was used to apply two-way ANOVA on the data.

### 2.9. MaxEnt Modeling

We downloaded future climate data predictions of 19 bioclimatic variables (Table S6) from WorldClim 1.4 at 30-arc second resolution (0.93 × 0.93 km = 0.86 km^2^ at the equator) [38] for ACCESS1.0 and CCSM4 models for the years 2050 and 2070 to representative concentration pathways (RCP), predicting urrence date in Asiad 4.5 and 8.5 climate change scenario [39]. The downloaded variable files were converted into ASC file format through QGIS 3.16.11. Bioclimatic variables were clipped onto the shapefile of Asia. These files were loaded on MaxEnt software version 3.4.4 and ran on the software [40]. Accuracy assessments for all models were randomly divided into 25% and 75% to conduct random tests and train the model. Background environmental data were applied by setting the background points maximum to 10,000 [41]. The model parameters were set to 5 replicates, and results were obtained from the average of all replicates of each model.

## 3. Results

**Figure 1:**
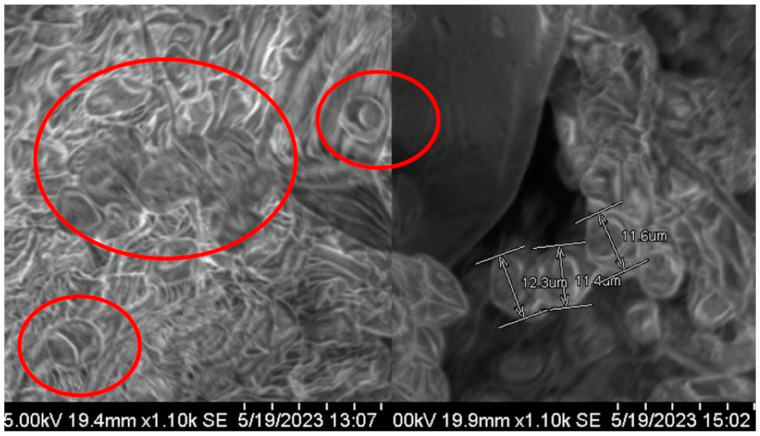
SEM images of *B. papyrifera* pollen captured at 50 µm resolution. Red circles indicate individual pollen, and double arrow lines show pollen diameter.

**Figure 2:**
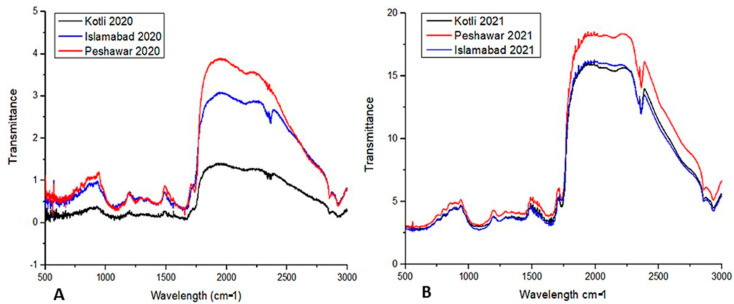
Inter-regional pollen FTIR analysis of *B. papyrifera*. Red, blue, and black lines denote R1, R2, and R3. (**A**) Pollen spectra of samples collected in the spring of 2020. (**B**) Pollen spectra of samples collected in the spring of 2021.

SEM analysis showed tectate exines and horizontal apertures of pollen. Pollen diameter was 6–12 µm on 50 µm resolution. Pollen from different regions have variations in pollen diameter (Figure 1) and FTIR spectra (Figure 2). Pollen FTIR analysis showed variations in the protein (amide-II and amide-I) and lipid region. The environmental conditions of these regions varied in terms of mean annual temperature and mean annual precipitation (see Figures S1 and S2). The data analysis showed a gradual increase in mean annual temperature and a gradual decrease in mean annual precipitation across all regions. The LC-MS/MS analysis of pollen sampled in 2020 showed 33 different organic compounds. These compounds were from six organic groups. Alkaloids were abundant, terpenes, alkanes, and straight-chain fatty acids were the lowest in the pollen samples.

All pollen grains have tectate exine surfaces. R1, R2, and R3 pollen have diameters of 6–12 µm calculated through SEM imaging scale at 50 µm resolution. For each region, 50 pollen SEM images are averaged to show pollen grain diameter (Table 1).

### 3.1. Fourier Transform Infrared Spectroscopy (FTIR) of Pollen

FTIR analysis of pollen (Figure 2A) collected from R1, R2, and R3 in spring 2020 using a spectral wavelength of 500–3000 nm showed spectral regions of the protein (1700–1500 cm^−1^) and lipid (2900–2700 cm^−1^) were measured. The region between 1750 cm^−1^ and 2750 cm^−1^ exhibited greater differences than other spectral regions. Figure 2B shows FTIR spectra of pollen samples collected from R1, R2, and R3 in spring 2020 and 2021. The R1, R2, and R3 pollen spectra vary for percent transmittance values. Spring 2020 pollen spectra peak heights are different than spring 2021 pollen spectra peak heights. The spectral differences associated with chemical bonds for proteins and lipids are listed in Table 2 (see Table 2).

The table shows spectral zones of lipids and proteins along with peak frequencies and chemical bonds [31].

**Figure 3:**
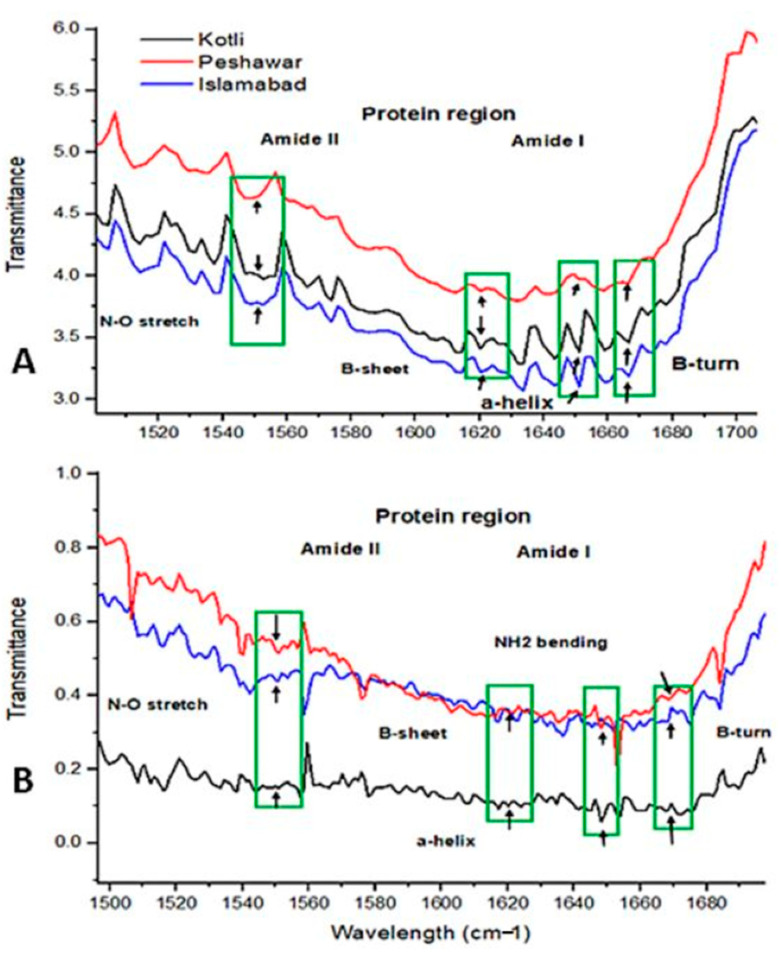
Location effect on FTIR spectra of pollen protein content collected in the spring 2020 and spring 2021: (**A**) Protein spectra of R1, R2, and R3 for pollen collected in 2020. (**B**) Protein spectra of R1, R2, and R3 pollen sampled in spring 2021. Green rectangles and black arrows show functional group comparisons.

*B. papyrifera* pollen collected from natural environments was analyzed by FTIR, which showed differences in protein profiles for R1, R2, and R3 (Figure 3A,B). Spectral differences in peak heights occur in Amide-II and Amide-I functional groups (1500–1700 wavelength). Amide-II region spectral peaks differ for all sites pollen at 1550 cm^−1^ wavelength in transmittance for N-O stretch. Similarly, the spectral peaks differ at 1620 cm^−1^ β-sheet, 1650 α-helix, and 1670 β-turn of the NH_2_ functional group in 2020 and 2021. R2 and R3 have sharp peaks N-O stretch at 1550 cm^−1^, 1620 cm^−1^ β-sheet, 1650 α-helix, and 1670 β-turn (NH_2_ functional group) in 2021 and 2020. The peaks for these wavelengths differ between spring 2021 and spring 2020 pollen for all sites.

**Figure 4:**
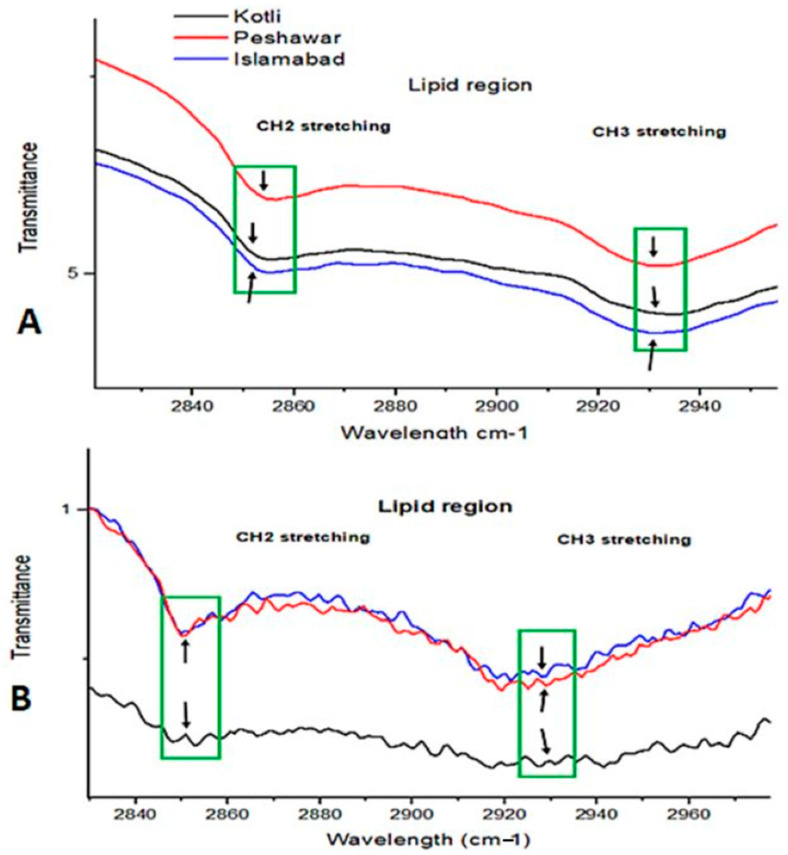
Seasonal and climatic effects on lipid region of pollen: (**A**) Spring 2020 pollen FTIR spectra for lipid region 2840 cm^−1^ to 2940 cm^−1^. (**B**) Spring 2021 pollen FTIR spectra for lipid region 2840 cm^−1^ to 2940 cm^−1^. Green rectangles and black arrows show functional group comparisons.

The lipid region had variation in peak intensities in 2020 for CH_2_ and CH_3_ functional groups at 2850 cm^−1^ and 2930 cm^−1^ for R1, R2, and R3 (Figure 4A,B). Pollen in the same region lost peak intensities in 2021. These variations in spectral peaks reflect differences in climatic variables’ impact on pollen structures.

### 3.2. Light Chromatography–Mass Spectrometry (LC-MS/MS) Analysis

**Figure 5:**
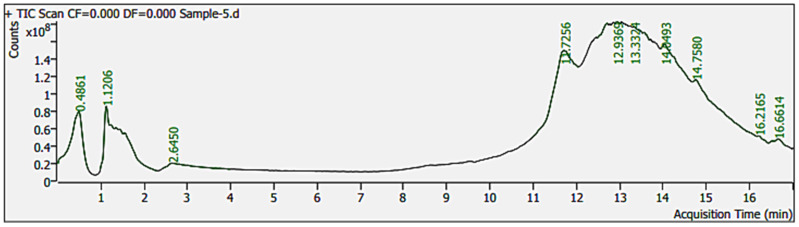
LC-MS/MS analysis of *B. papyrifera* pollen. The Y-axis shows charge to mass ratio for the ions of various compounds separated at different time intervals, while the X-axis denotes acquisition time in minutes. The sample run time is 17 min, with the highest peak at 13th minutes 12.9369. The analysis shows the variance in charge-to-mass ratios of ions in the pollen that make unique peaks. Peaks analysis reveals the presence of different compounds in the analyzed pollen (for details, see Table S4).

**Figure 6:**
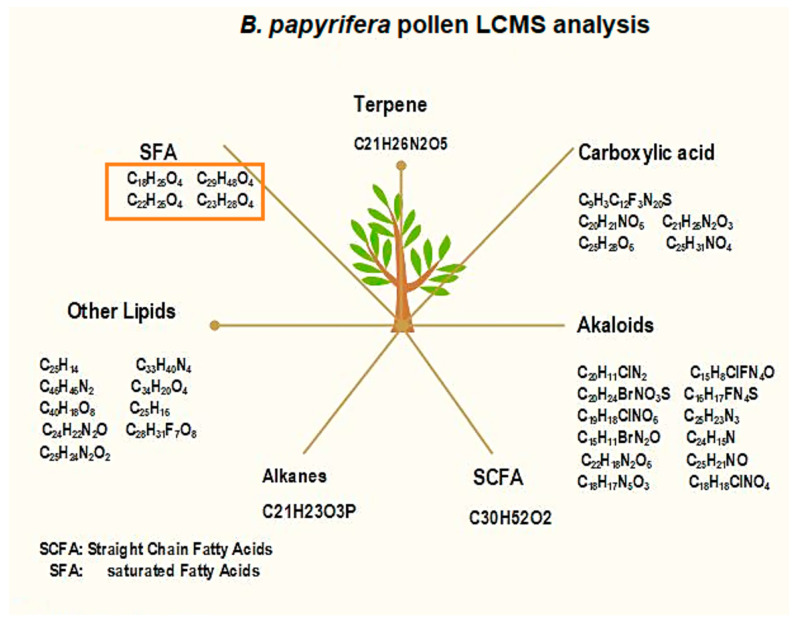
Schematic distribution of organic compounds in *B. papyrifera* pollen grains by LC-MS/MS analysis. Total compounds identified from the NIST database in the pollen samples were classified into 7 groups: alkanes, alkaloids, carboxylic acids, terpenes, other lipids, straight-chain fatty acids, and unsaturated fatty acids. Unsaturated fatty acids (marked in orange show the presence of allergenic compounds in the pollen grains of *B. papyrifera*.

LC-MS/MS analysis of *B. papyrifera*-pollen run time was 17 min (Figure 5). LC-MS/MS analysis identified many compounds, as shown in Table S4. These compounds were categorized into seven groups, including alkaloids, carboxylic acids, alkanes, straight-chain fatty acids, terpenes, unsaturated fatty acids (USFA), and other lipids, as shown in Figure 6. Alkaloids occur in the highest numbers (12), followed by other lipids (9) and carboxylic acids (5). Alkanes, straight-chain fatty acids, and terpenes occur very little in quantity. There was only one straight-chain fatty acid (C_30_H_52_O_2_), one alkene (C_21_H_23_O_3_P), and one terpene (C_21_H_26_N_2_O_5_). Saturated fatty acids identified in this study include—Succinic acid, hexyl 2-phenylethyl ester (C_18_H_26_O_4_), Adipic acid, di (2-phenylethyl) ester (C_22_H_26_O_4_), Pimelic acid, di(phenethyl) ester (C_23_H_28_O_4_), and Sebacic acid, 2-methylbenzyl undecyl ester (C_29_H_48_O_4_) in the target pollen.

### 3.3. In Vitro IgE Binding to B. papyrifera Pollen Proteins

**Figure 7:**
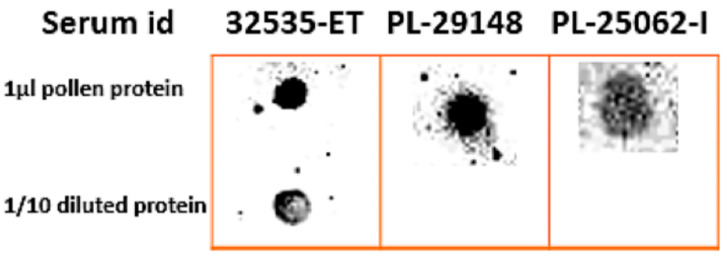
Dot blot of *B. papyrifera* pollen-protein extract. Samples of 1 µL of pure pollen-protein extracts and 1/10 diluted pollen-protein extracts in 1X PBS blotted on a nitrocellulose paper against 1:1000 diluted anti-IgE monoclonal HRP Southern Biotech antibodies. The dark spots show IgE binding with the respective serum ID given above the spot.

Selected 13 serum 1:10 diluted samples in non-fat dry milk were cross-reacted with *B. papyrifera* pollen protein extracts in 1X PBS and 1:10 (1X PBS) diluted through dot blots. Three serum samples showed IgE binding with 1 µL pollen protein, and only one serum sample (32535-ET) showed IgE binding at 1:10 dilution. These three positive serum samples (Figure 7) were shortlisted for Western blotting to identify allergens in the pollen protein extracts.

**Figure 8:**
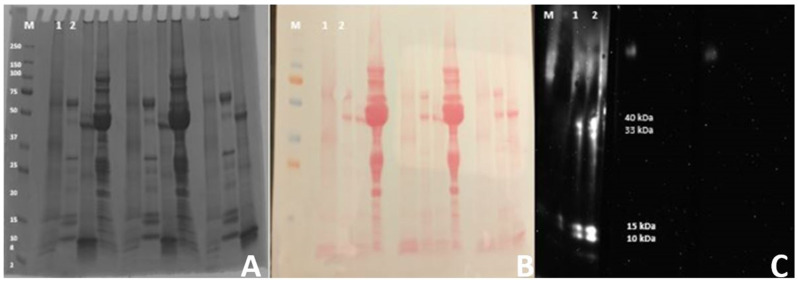
Western blot analysis of *B. papyrifera*-pollen protein: (**A**) *B. papyrifera*-pollen protein on a stained gel with 250 kDa pre-stained Precision Plus molecular weight marker proteins denoted as M, lane 1, and lane 2 shows *B. papyrifera*-pollen proteins. (**B**) Western blotting image of proteins on the nitrocellulose membrane stained with safranin red. (**C**) Anti-IgE binding with allergens on Western blot. The Western blot analysis depicts 4 allergen bands in lane 1 and lane 2, with primary antibody binding with 1:1000 diluted secondary antibodies.

Allergenic proteins in the selected sera were determined through SDS gel electrophoresis and Western blotting. Western blotting identified four protein bands in lane 1 and lane 2 (see Figure 8). The band sizes 40 kDa, 33 kDa, 15 kDa, and 10 kDa were confirmed by comparison of the Western blot with the stained gel on the imager stage after Western blot visualization. Three protein bands of 40 kDa, 33 kDa, and 10 kDa were previously identified, while a 15 kDa protein band is not reported in any literature.

### 3.4. Precipitation, Temperature and Pollen Count

The three decades’ data analysis (1991–2020) for R1 and R2 and a single decade’s data (2010–2020) available from PMD showed a decrease in mean annual precipitation and an increase in mean annual temperature during this period for the selected three regions (see Figures S1 and S2). The trend line for this period shows about 13 mm mean annual precipitation decrease for R1, while the trend line for R2 and R3 shows a few mm mean annual precipitation decrease. Similarly, the temperature change trend line for all three regions showed an increase in the temperature for the mentioned period. About 0.45 °C increase in mean annual temperature has occurred for R1. About 0.2 °C increase in mean annual temperature has occurred in R2 and R3. Analysis of spring maximum single-day pollen count in 1 cubic meter in R2 found pollen count production increase during 2003–2022 (see Figure S5). The maximum single-day pollen count was 38,946 pollen grains/m^3^ in 2003, which reached the highest at 49,465 pollen grains/m^3^ in 2022.

### 3.5. Model Performance of the MaxEnt Model

**Figure 9:**
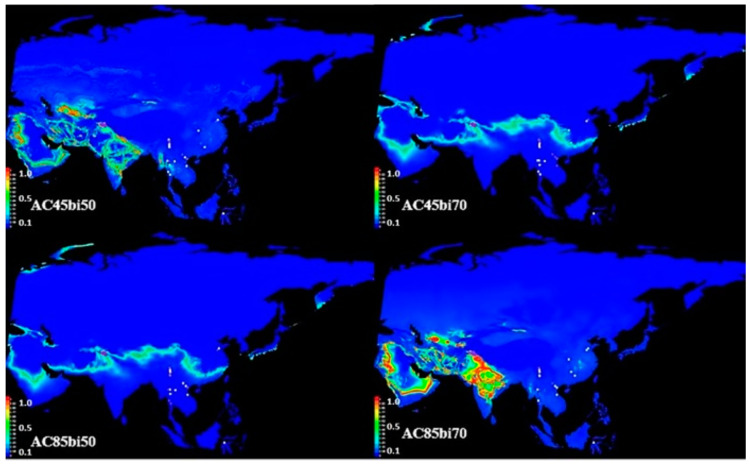
ACCESS1.0 based MaxEnt modeling of *B. papyrifera* growth occurrence in the years 2050 and 2070 in Asia. The figure shows habitat suitability for *B. papyrifera* in the years 2050 and 2070 through the ACCESS1.0 Coupled Model Intercomparison Project Phase 5 (CMIP5) dataset. AC represents ACCESS, 4.5 shows representative concentration pathway (RCP) 4.5, 8.5 shows representative concentration pathway (RCP) 8.5, and bi50 and bi70 denote bioclimatic variables in the year 2050 and 2070. The colored scale (0–1) on the right side of the map shows the suitability of locations for *B. papyrifera*-growth invasion in the future.

**Figure 10:**
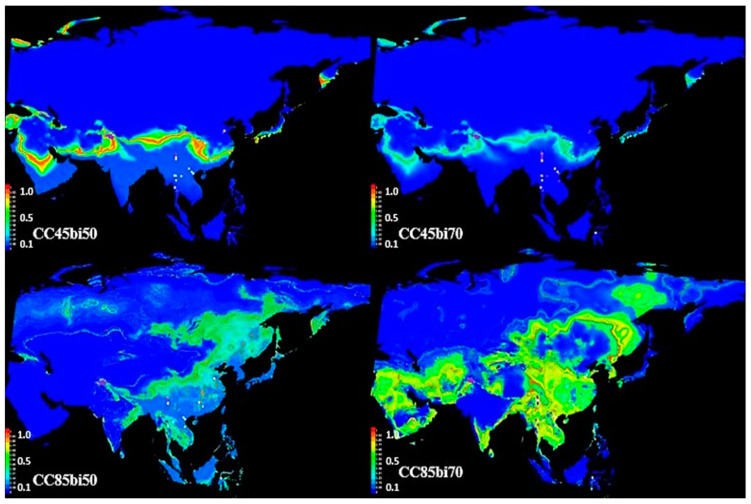
CCSM4-based MaxEnt modeling of *B. papyrifera* growth occurrence in the years 2050 and 2070 in Asia. The figure shows habitat suitability for *B. papyrifera* in the years 2050 and 2070 through the CCSM4 CMIP5 dataset. CC represents CCSM4, 4.5 shows representative concentration pathway (RCP) 4.5, 8.5 shows representative concentration pathway (RCP) 8.5, and bi50 and bi70 denote bioclimatic variables in the years 2050 and 2070. The colored scale (0–1) on the left side of the maps shows the suitability of locations for *B. papyrifera*-growth invasion in the future.

The area under the curve (AUC) is a built-in statistical package in MaxEnt that evaluates model performance based on the presence of records data only [42]. The AUC test exhibited 0.968, 0.99, 0.99, 0.938, 0.962, 0.987, 0.959, and 0.881 for training data of all models that showed excellent performance of the model in predicting the potential distribution of *B. papyrifera* in Asia in the years 2050 and 2070 under RCP 4.5 and RCP 8.5 (Figure 9 and Figure 10).

Out of 19 CMIP5 bioclimatic variables used in this study (Table S6), bioclimatic variables BIO701 (mean annual temperature), BIO7010 (mean temperature of warmest quarter), BIO7011 (mean temperature of coldest quarter), and BIO7012 (annual precipitation) have a significant contribution to the MaxEnt model analysis (Figure S6). The permutation importance of these four variables was the highest of all other variables that predict change in the values of these variables in the future will add to habitat suitability for plant growth. MaxEnt modeling through ACCESS1.0 for climate change scenario RCP 4.5 and RCP 8.5 shows a perspective increase in the occurrence of *B. papyrifera* in Asia. The increase occurs due to the mean annual temperature increase. AC4.5bi50 showed a greater increase in the possible habitat suitability of the tree in Saudi Arabia, Iraq, Jordan, Syria, Turkey, Iran, Afghanistan, Turkmenistan, Uzbekistan, Afghanistan, Pakistan, India, and Nepal, and a slight increase in China, Hong Kong, and Myanmar. The prospective invasion of *B. papyrifera* in model AC8.5bi50 is similar to AC4.5bi50, but the area covered in this model is greater, and the intensity of plant growth is also greater. Models AC4.5bi70 and AC8.5bi50, with almost similar temperature conditions, showed suitable growth conditions that can facilitate *B. papyrifera* invasion in regions of Saudi Arabia, Iran, Pakistan, India, Afghanistan, China, Nepal, Hong Kong, and Japan. Both RCP 4.5 and 8.5 scenarios in the years 2050 and 2070 show suitable habitats for *B. papyrifera* invasion into uninvaded regions of Asia.

Bioclimatic variables BIO701 (mean annual temperature), BIO7010 (mean temperature of warmest quarter), BIO7011 (mean temperature of coldest quarter), and BIO7012 (annual precipitation) contributed to the model’s future assessment analysis. The model has assessed that a change in the values of these three variables in the future will improve the habitat for *B. papyrifera* growth, which will facilitate plant invasion and plant growth in new locations in Asia. MaxEnt modeling through CCSM4 for climate change scenarios RCP 4.5 and RCP 8.5 shows the prospective increase in the occurrence of the *B. papyrifera* tree. CC4.5bi50 shows habitat suitability where the plant can invade in the future. The CC4.5bi50 model assessment for the tree is similar to AC4.5bi50 and AC4.5bi70, with a slight difference in the invasion intensity. However, CC8.5bi50 showed a prospective habitat for the tree invasion in almost half of Asia in the year 2050, while the tree invasion will continue in Asia by 2070, according to CC8.5bi70. Both RCP 4.5 and 8.5 scenarios in the years 2050 and 2070 show the greater invasion of the tree into uninvaded regions in the years 2050 and 2070. Both models (Access 1.0 and CCSM4) predict habitat suitability for *B. papyrifera,* which can invade new locations in Asia in the years 2050 and 2070 climate change scenarios RCP 4.5 and RCP 8.5. However, the CCSM4 model predicts high habitat suitability for species invasion as compared to the ACCESS1.0 model in 2050 and 2070 under RCP 4.5 and RCP 8.5.

## 4. Discussion

The current research showed *B. papyrifera* pollen size/diameter (6–12 µm) through SEM analysis (Figure 1). Polysciences, a commercial pollen vendor, reported that the plant pollen size is 12–13 um, which is similar to our study’s results [43]. SEM analysis did not find the varied temperature, precipitation, and elevation effects on *B. papyrifera* pollen’s morphology. However, FTIR analysis showed differences in pollen biochemical composition through spectral peaks between 4000 cm^−1^ and 400 cm^−1^ (Figure 2, Figure 3 and Figure 4). FTIR spectral peak differences can be due to changes in the functional groups of biomolecules making pollen. Pollen structure comprises lipids, carbohydrates, and proteins [44,45,46,47,48]. The *B. papyrifera* pollen FTIR analysis for protein and lipid functional groups showed differences in R1, R2, and R3 pollen grains. The spring 2020 pollen spectra varied in the protein region (1500 cm^−1^–1700 cm^−1^). Amide I region and Amide II region peaks differed at 1620 cm^−1^, 1650 cm^−1^, 1670 cm^−1^, and 1550 cm^−1^ for β-sheet, α-helix, β-turn, and NO-stretch (Figure 3A,B). Peak areas in the Amide I region for β-sheet, α-Helix, and β-turn are similar for R2 and R3 and vary for R1. FTIR spectral peaks for CH_2_ and CH_3_ lipid functional groups vary for spring 2020 and 2021 pollens (Figure 4A,B). These FTIR results are in good agreement with the previous findings, which showed temperature and precipitation effects on pollen chemical composition and viability of *Syringa vulgaris* L. and *Phleum pratense* [28,33]. They found spectral differences in pollen of different species. Our findings show spectral differences in *B. papyrifera* pollen (protein and lipid region) collected from the three locations at varied temperatures and precipitation conditions. These inter-regional differences in the protein region of FTIR peaks can be correlated to variations in temperature and precipitation conditions of R1, R2, and R3 (Figures S1, S2 and Figure 3).

The mean annual temperature of R1 is higher than R2 and R1, and the mean annual precipitation of R1 is lower than R2 and R3 (Figure S2). The spectra analyzed for the three regions showed elevated peaks and intense transmission for R1 as compared to R2 and R3. This enhancement in the spectra for R1 can be due to R1 bioclimatic variables and geographic location. Similar findings were made for hazel interregional pollen comparison through FTIR spectroscopy. Such variations in pollen biochemical structure are adaptive to bioclimatic variable differences [31]. High-temperature exposure reduces in vitro pollen germination percentage and pollen-tube length in *Pisum sativum* L. [12]. High-temperature stress to pollen yields an increase in pollen proteins, while low-temperature stress causes enhancement of pollen lipids and carbohydrates [30,49]. Similarly, an increase in temperature and a decrease in precipitation affected pollen weight, protein content, and allergen contents [2]. High-temperature stress causes morpho-anatomical changes in pollen and its reproductive failure [10]. Comparably, FTIR spectroscopy identified adaptive variations in the chemical composition of proteins, lipids, and carbohydrates in pollen of *Poa alpina*, *Anthoxanthum odoratum*, and *Festuca ovina* grown in different geographic and climatic conditions [30]. Those investigations supported increases in temperature and elevation differences alter pollen chemical composition. Moreover, variations in environmental conditions have altered lipids, carbohydrates, and protein expression in 813 pollen specimens from 300 distinct plant species grown in five different pollen seasons [29].

Our investigations on *B. papyrifera* pollen’ metabolome analysis through LC-MS/MS have found the presence of succinic acid, hexyl 2-phenylethyl ester (C_18_H_26_O_4_); adipic acid, di (2-phenylethyl) ester (C_22_H_26_O_4_); pimelic acid, di (phenethyl) ester (C_23_H_28_O_4_); and sebacic acid, 2-methylbenzyl undecyl ester (C_29_H_48_O_4_). Four lipid compounds (succinic acid, adipic acid, pimelic acid, and sebacic acid) have previously been reported to have a role in pollen allergies of some subjects [50,51]. These compounds’ role was found in activating cytokine expression of DC/NKT cells and were marked as potential biomarkers [50]. These saturated fatty acids can induce T Helper 2 (TH2) cells associated with Interleukine-13 (IL-13) that produce stimulatory responses in humans. Exposure of epithelial layers of the nasal cavity to allergenic pollen (containing allergens and allergenic metabolites) elicits the human immune system.

This study reports four potential allergens in *B. papyrifera* pollen, causing pollen allergy in susceptible human populations. Previously, three allergens, 33 kDa, 40 kDa, and 10 kDa, were reported in *B. papyrifera* pollen through ELISA and blotting studies [20,52]. This study confirms the presence of 33 kDa, 40 kDa, and 10 kDa allergens in the *B. papyrifera* pollen and finds one novel allergen of about 15 kDa through Western blotting. Four *Moraceae* family allergens are recorded in Allergenonline.org (accessed on 7 January 2023) and Allergen.org (accessed on 7 January 2023) databases. Out of these four, Mor n 3 (non-specific lipid transfer protein) and two other 18 kDa unassigned are food allergens. Mor a 2 (cobalamin-independent methionine synthase) is the only registered airway allergen of 84 kDa in the allergen.org database. This study found a 15 kDa putative novel allergen and verified three previously identified airway allergens in *B. papyrifera* pollen. None of the four allergens identified in this study are registered in Allergenonline.org and Allergen.org. Historical pollen dataset analysis showed an increase in the spring single-day maximum pollen count trend (Figure S5). *B. papyrifera* pollen increase trend in the plant growing regions makes the susceptible population more vulnerable to pollen allergy. It is expected that *B. papyrifera* pollen allergy will increase in the future due to high habitat suitability for plant growth and distribution.

RCP 4.5 estimates a moderate increase in global temperature and carbon availability in the atmosphere [53]. MaxEnt modeling, according to RCP 4.5, estimates greater habitat suitability for *B. papyrifera* growth in new regions of Asia (Figure 9). Similarly, an extreme increase in global temperature and carbon emissions, according to RCP 8.5 [54], assessed habitat suitability through MaxEnt modeling, which suggests *B. papyrifera* invasion into uninvaded regions of Asia in the future (Figure 10). ACCESS1.0 and CCSM4 models through Maxent estimated habitat suitability for the invasion of the allergenic species into new locations. Further, analysis of bioclimatic variables [mean annual temperature (BIO701), mean temperature of warmest quarter (BIO7010), mean temperature of coldest quarter (BIO7011), and BIO7012 (annual precipitation)] percent contribution and permutation to the two models assessed their role in *B. papyrifera* invasion. An increase in BIO1, BIO10, and BIO11 variable values in the future due to climate change contributes to the model output. The model output correlated the tree growth habitat suitability with mean annual precipitation and temperature. Similarly, changes in the pollen metabolome correlated with changes in mean annual temperature and precipitation. An increase in these variables values in the future occurs due to increase in carbon emissions [55]. The expected increase in the habitat suitability for *B. papyrifera* growth can facilitate its invasion into new regions. *B. papyrifera* invasion will expose the susceptible human population to the allergenic pollen that may invoke immune reactions. Similarly, ragweed pollen allergy 2041–2060 assessment in Europe found that the plant invasion will cause pollen sensitization in 33 to 77 million people [56].

## 5. Conclusions

Our state-of-the-art investigations have found a putative novel airway allergen (about 15 kDa) in *B. papyrifera* pollen through Western blotting and verified the presence of three other allergens reported earlier. So far, none of these four airway allergens are registered in Allergenonline.org and Allergen.org. The study’s findings demonstrate that factors like temperature, precipitation, and differential elevation may have affected *B. papyrifera* pollen biochemical composition in the R1, R2, and R3 regions. Among 33 organic compounds identified by LC-MS/MS analysis, four saturated fatty acid compounds have a possible role in eliciting cytokine expression of DC/NKT cells in the human immune system. *B. papyrifera* MaxEnt modeling estimated the habitat suitability for the plant growth in Asia in the years 2050 and 2070 can enhance the plant pollen allergy. The study findings provide a baseline model system for studying other allergenic plant species’ pollen metabolome, predicting their growth invasion, and associated allergies in response to climate shift.

## Figures and Tables

**Table 1:** SEM data of pollen grain average diameter.

Image Name	Location and Time	Average Pollen Grain Diameter µm	Standard Deviation
i	Spring 2020 Peshawar (R1)	7.04	±0.61
ii	Spring 2020 Islamabad (R2)	8.86	±0.68
iii	Spring 2020 Kotli (R3)	10.02	±0.38
iv	Spring 2021 Peshawar (R1)	6.64	±0.36
v	Spring 2021 Islamabad (R2)	11.02	±0.62
vi	Spring 2021 Kotli (R3)	11.5	±0.26

**Table 2:** Spectral zone, functional group frequencies, and associated chemical bonds.

Spectral Zone	Peak Frequency cm^−1^	Chemical Bonds
Lipids	2930	CH_3_ stretching (lipids)
	2850	CH_2_ stretching (lipids)
Amide I	1620 β-sheet	
1700–1600 cm^−1^	1650 α-helix	NH_2_ bending
	1670 β-turn	
Amide II	1550	N-O stretch
1600–1500 cm^−1^		

## Data Availability

The original contributions presented in this study are included in the article/Supplementary Materials. Further inquiries can be directed to the corresponding author.

## Supplementary Materials

The following supporting information can be downloaded at: https://www.mdpi.com/article/10.3390/metabo15020137/s1, Figure S1. Mean annual precipitation of R1, R2, and R3 from 2010 to 2020 and their two-way ANOVA. Black, red, and green colors represent R1, R2, and R3. The line graph shows the mean annual precipitation, and the bar graph shows their two-way ANOVA. Figure S2. Mean annual temperature of R1, R2, and R3 from 2010 to 2020 and their two-way ANOVA. Black, red, and green colors represent R1, R2, and R3. The line graph shows the mean annual temperature, and the bar graph shows their two-way ANOVA. Figure S3. Map of Pakistan showing *B. papyrifera* pollen sampling regions. The map shows regions R1, R2, and R3, where *B. papyrifera* pollen was sampled in spring 2020 and spring 2021. Pollen was collected from three nearby trees from each region. Figure S4. *B. papyrifera* pollen collection in the study regions. Figure S5. *B. papyrifera* pollen count from 2002 to 2022. Figure S6. Individual bioclimatic variables contribution to *B. papyrifera* MaxEnt modeling. Table S1. Mean annual precipitation and mean annual temperature data were obtained from PMD through email requests. Table S2. *B. papyrifera* occurrence in Asia sheet in csv file format. The Excel sheet contains *B. papyrifera* occurrence in Asia obtained from GBIF.org and field observation in Pakistan. Table S3. *B. papyrifera* sampling sheet. The sheet contains pollen sampling information from R1, R2, and R3 in spring 2020 and spring 2021. Table S4. *B. papyrifera* pollen LC-MS/MS analysis results. Table S5. Sera purchased from PLASMA Lab USA in August–November 2022 allergic to *B. papyrifera* pollen. Table S6. Bioclimatic variables downloaded from Worldclim.org at 30 s resolutions.
