# Supplementary material for: Broussonetia papyrifera Pollen Metabolome Insights, Allergenicity, and Dispersal in Response to Climate Change Variables
Source: Metabolites. 2025 Feb 18;15(2):137. doi: 10.3390/metabo15020137 (PMC11857163; doi:10.3390/metabo15020137)
Supplement: Supplementary file 1 [file metabolites-15-00137-s001.zip › S4 Table..pdf]

Unknown Analysis Report - All Hits

**Batch Path**	D:\MassHunter\Data\Pesticide\Phenol\12-11-2020		
**Analysis File Name**	12-11-2020.uaf		
**Analyst Name**	LCMSMS		
**Analysis Time**	12/11/2020 3:30:54 PM		
**Data File Name**	Sample-5.d	**Data Path Name**	D:\MassHunter\Data\Pesticide\Phenol\12-11-2020
**Sample Name**	Sample-5	**Sample Type**	Sample
**Acq Method File**	Phenolic Acid.m	**Acq Method Path**	D:\MassHunter\Methods
**Acq Time**	12/11/2020 12:03:10 PM	**Operator**	
**Instrument Name**	Agilent Triple Quad	**Dilution**	1

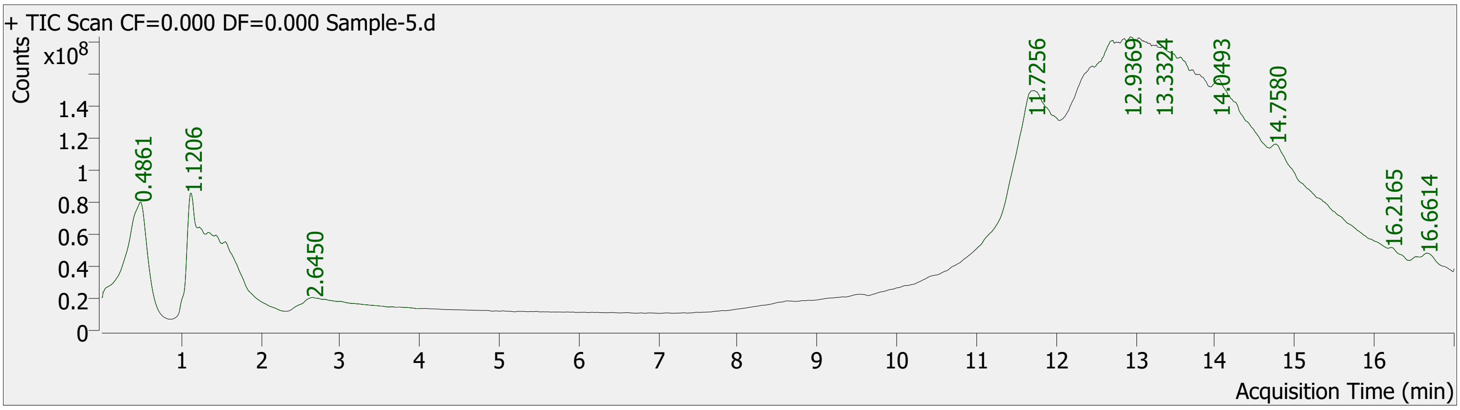

RT	Compound Name	CAS#	Formula	Area	Match Score	Sample	Sample
0.4861	Coronene, methyl-	13119-86-3	C25H14	1365892813	54.2	6.62	32.26
0.4861	11-Chlorodibenzo(a,c)phenazine	4618-89-7	C20H11ClN2	1365892813	54.0	6.62	32.26
0.4861	4-Chloro-2-{9-fluoro-[1,2,4]triazolo[1,5-c]quinazolin-2-yl}phenol	1000435-69-4	C15H8ClFN4O	1365892813	51.7	6.62	32.26
1.1206	2-Phenylethyl docosanoate	104899-74-3	C30H52O2	561659522	69.0	2.72	13.26
1.1206	Succinic acid, hexyl 2-phenylethyl ester	1000325-03-7	C18H26O4	561659522	67.9	2.72	13.26
1.1206	Succinic acid, octyl 2-phenylethyl ester	1000325-03-9	C20H30O4	561659522	67.1	2.72	13.26
1.2277	Zirconium, dichloro-[dimethylbis(2-methyl-4-phenylindenyl)silane]	1000164-77-3	C34H30Cl2SiZr	300295597	57.8	1.46	7.09
1.2277	2,2':3',2'':3'',2'''-Quaternaphthalene-1,1',1'',1''',4,4',4'',4'''-octone	125520-49-2	C40H18O8	300295597	42.7	1.46	7.09
1.2277	benzenemethanamine, N,N'-(cyclohexylidenedi-4,1-phenylene)bis[N'-(phenylmethyl)-	1000402-99-8	C46H46N2	300295597	42.5	1.46	7.09
1.3348	2-Amino-3-(3,4-dimethoxyphenyl)-4H-benzo[g]chromen-4-one ditms	1000331-93-2	C27H33NO4Si2	309577549	46.3	1.50	7.31
1.3348	1,4-Bis(2-naphthyloxy)anthraquinone	1000110-90-5	C34H20O4	309577549	45.3	1.50	7.31
1.3348	2,8,10,12,18-Pentamethyl-3,7,13,17-tetraethyl-21H,23H-porphine	105043-42-3	C33H40N4	309577549	43.8	1.50	7.31
1.4337	2-Phenylethyl docosanoate	104899-74-3	C30H52O2	289891823	62.8	1.40	6.85
1.4337	Adipic acid, di(2-phenylethyl) ester	1000324-58-0	C22H26O4	289891823	62.1	1.40	6.85
1.4337	Pimelic acid, di(phenethyl) ester	1000416-50-6	C23H28O4	289891823	61.9	1.40	6.85
1.5573	2-(2-Fluoro-6a,8a,10,10-tetramethyl-4-oxo-7-(2,2,2-trifluoroacetoxy)-2,4,5,6,6a,6b,7,8,8a,8b,11a,12,12a,12b-tetradecahydro-1H-naphtho[2',1':4,5]indeno[1,2-d][1,3]dioxol-8b-yl)-2-oxoethyl 2,2,2-trifluoroacetate	1000385-76-0	C28H31F7O8	720017745	42.7	3.49	17.00
1.5573	(S)-7-Bromo-3-isobutyl-2-(2-methylbenzyl)-3,4-dihydro-2H-benzo[b][1,4,5]oxathiazepine 1,1-dioxide	1233355-16-2	C20H24BrNO3S	720017745	42.2	3.49	17.00
1.5573	Sebacic acid, 2-methylbenzyl undecyl ester	1000380-72-1	C29H48O4	720017745	39.2	3.49	17.00
2.6450	4-(2-Chloro-phenyl)-1-cyclopropyl-1,4-dihydro-pyridine-3,5-dicarboxylic acid dimethyl ester	1000275-22-1	C18H18ClNO4	241504509	49.5	1.17	5.70
2.6450	4-(6-Chloro-benzo[1,3]dioxol-5-yl)-1-cyclopropyl-1,4-dihydro-pyridine-3,5-dicarboxylic acid dimethyl ester	1000275-03-3	C19H18ClNO6	241504509	48.6	1.17	5.70
2.6450	1-Cyclopropyl-4-(4-methoxycarbonyl-phenyl)-1,4-dihydro-pyridine-3,5-dicarboxylic acid dimethyl ester	1000275-22-2	C20H21NO6	241504509	45.5	1.17	5.70
11.7256	N-(5,6-Dichloro-1,3-benzothiazol-2-yl)-2,2,2-trifluoroacetamide	1000373-29-0	C9H3Cl2F3N2OS	3331528237	42.3	16.15	78.67
11.7256	(1H)Benzimidazole, 5-fluoro-2-(2-thienyl)-6-(4-methylpiperazin-1-yl)-	174468-69-0	C16H17FN4S	3331528237	38.3	16.15	78.67
11.7256	6-Bromo-2-methoxy-4-phenyl-quinazoline	1000318-41-9	C15H11BrN2O	3331528237	37.5	16.15	78.67
12.4590	11-Chlorodibenzo(a,c)phenazine	4618-89-7	C20H11ClN2	2222681794	43.2	10.77	52.49

RT	Compound Name	CAS#	Formula	Area	Match Score	Sample	Sample
12.4590	Coronene, methyl-	13119-86-3	C25H14	2222681794	41.1	10.77	52.49
12.4590	5'-Fluoro-2'-(tert.-butyldimethylsilyl)oxy-4-methylchalcone	1000454-17-9	C22H27FO2Si	2222681794	40.7	10.77	52.49
12.7144	Zirconium, dichloro-[dimethylbis(2-methyl-4-phenylindenyl)silane]	1000164-77-3	C34H30Cl2SiZr	1502170002	55.5	7.28	35.47
12.7144	Yohimban-16-carboxylic acid, 17-hydroxy-, methyl ester, (16.beta.,17.alpha.)-	483-10-3	C21H26N2O3	1502170002	43.7	7.28	35.47
12.7144	Gallacetophenone-4'-methylether, bis(tert-butyldimethylsilyl) ether	1000462-95-3	C21H38O4Si2	1502170002	43.2	7.28	35.47
12.7556	11-Chlorodibenzo(a,c)phenazine	4618-89-7	C20H11ClN2	468472118	48.5	2.27	11.06
12.7556	Coronene, methyl-	13119-86-3	C25H14	468472118	48.1	2.27	11.06
12.7556	7-Bromo-5-chloro-8-hydroxyquinoline, tert-butyldimethylsilyl ether	1000463-53-6	C15H19BrClNOSi	468472118	46.8	2.27	11.06
12.8627	Coronene, methyl-	13119-86-3	C25H14	520718585	55.1	2.52	12.30
12.8627	5'-Fluoro-2'-(tert.-butyldimethylsilyl)oxy-4-methylchalcone	1000454-17-9	C22H27FO2Si	520718585	52.4	2.52	12.30
12.8627	11-Chlorodibenzo(a,c)phenazine	4618-89-7	C20H11ClN2	520718585	51.5	2.52	12.30
12.9369	9,9'-Spirobifluorene	1000326-43-1	C25H16	1076500291	43.5	5.22	25.42
12.9369	6H-Benzo[b]naphtho[2,3-h]carbazole	905-95-3	C24H15N	1076500291	42.7	5.22	25.42
12.9369	15H-Benzo[a]naphtho[2,3-h]carbazole	17182-04-6	C24H15N	1076500291	41.7	5.22	25.42
13.0358	11-Chlorodibenzo(a,c)phenazine	4618-89-7	C20H11ClN2	2544429052	49.0	12.33	60.09
13.0358	7-Bromo-5-chloro-8-hydroxyquinoline, tert-butyldimethylsilyl ether	1000463-53-6	C15H19BrClNOSi	2544429052	46.5	12.33	60.09
13.0358	9,9'-Spirobifluorene	1000326-43-1	C25H16	2544429052	46.3	12.33	60.09
13.3324	5'-Fluoro-2'-(tert.-butyldimethylsilyl)oxy-4-methylchalcone	1000454-17-9	C22H27FO2Si	1654231499	46.2	8.02	39.06
13.3324	Estra-1,3,5(10)-trien-17-one, 3-(acetyloxy)-4-nitro-, 17-(O-methyloxime)	77883-07-9	C21H26N2O5	1654231499	44.7	8.02	39.06
13.3324	Tungsten, [(1,2,3-.eta.)-2-methyl-2-propenyl]tris(.eta.3-2-propenyl)-	127629-47-4	C13H22W	1654231499	43.8	8.02	39.06
13.5631	Bis(.mu.-propynyl)-bis(cyclopentadienyl)zirconium-bis(t-butylcyclopentadienyl)zirconium	1000154-45-0	C34H42Zr2	1259765549	50.0	6.11	29.75
13.5631	5'-Fluoro-2'-(tert.-butyldimethylsilyl)oxy-4-methylchalcone	1000454-17-9	C22H27FO2Si	1259765549	46.1	6.11	29.75
13.5631	1-(4-Hydroxyphenyl)-4-(4-chlorophenyl)-(1H)-pyrimidin-2-thione	1000286-95-8	C16H11ClN2OS	1259765549	43.0	6.11	29.75
13.7032	7-Bromo-5-chloro-8-hydroxyquinoline, tert-butyldimethylsilyl ether	1000463-53-6	C15H19BrClNOSi	1864120693	44.7	9.03	44.02
13.7032	11-Chlorodibenzo(a,c)phenazine	4618-89-7	C20H11ClN2	1864120693	43.2	9.03	44.02
13.7032	7-Bromo-5-chloro-8-hydroxyquinoline, trimethylsilyl ether	1000463-53-9	C12H13BrClNOSi	1864120693	43.0	9.03	44.02
14.0493	Silane, diethylheptadecyloxy(2-methoxyethoxy)-	1000363-54-6	C24H52O3Si	4234649045	46.3	20.52	100.00
14.0493	acetic acid, 2,2'-[(2,2',3,3'-tetrahydro-3,3,3',3'-tetramethyl-1,1'-spirobi[1H-indene]-6,6'-diyl)bis(oxy)]bis-	1000399-17-5	C25H28O6	4234649045	44.8	20.52	100.00
14.0493	Cinnamic acid, .alpha.-[N-benzoylamino]-3,5-di-t-butyl-4-hydroxy-	95820-15-8	C25H31NO4	4234649045	44.1	20.52	100.00
14.7580	Dioxybenzone, bis(tert-butyldimethylsilyl)-	1000453-70-1	C26H40O4Si2	3528779615	52.4	17.10	83.33
14.7580	Silane, diethyl(2-ethoxyethyloxy)octadecyloxy-	1000363-52-8	C26H56O3Si	3528779615	49.5	17.10	83.33
14.7580	3-(Tert.-butyldimethylsilyl)oxy-7,8,2',3'-tetramethoxyflavone	1000454-18-2	C25H32O7Si	3528779615	48.1	17.10	83.33
16.2165	8-(4-Methoxyphenyl)-11,11-dimethyl-8,10,11,12-tetrahydrobenzo[a][4,7]phenanthrolin-9(7H)-one	1000263-38-0	C25H24N2O2	128266688	39.0	0.62	3.03
16.2165	Dimethoxymethyl-hydroxy-triphenyl phosphide	1000132-00-1	C21H23O3P	128266688	36.3	0.62	3.03
16.2165	Dibenzo[c,f]1,7-naphthyridin-9(7H)-one, 8,10,11,12-tetrahydro-11,11-dimethyl-8-phenyl-	1000271-48-2	C24H22N2O	128266688	34.9	0.62	3.03
16.5461	4-(4-Acetylamino)-2,6-diphenylpyrimidine	130090-19-6	C24H19N3O	37095007	42.7	0.18	0.88
16.5461	2,5-piperazinedione, 3,6-bis[[4-(acetyloxy)phenyl]methylene]-	1000402-74-5	C22H18N2O6	37095007	41.9	0.18	0.88
16.5461	1-Methyl-2,6-diphenyl-4,4-pentamethylene-1,4-dihydropyridine-3,5-dicarbonitrile	83078-31-3	C25H23N3	37095007	39.0	0.18	0.88
16.6614	Cyclodisilazane, 2,2,4,4-tetramethyl-1,3-bis(methyldiphenylsilyl)-	21116-67-6	C30H38N2Si4	91307132	34.6	0.44	2.16
16.6614	6-Acetyl-1,3,8-trimethyl-7-phenyl-1H-imidazo[2,1-f]-purine-2,2(3H,8H)-dione	72793-12-5	C18H17N5O3	91307132	31.4	0.44	2.16
16.6614	.alpha.-Pyridone, 3,5-dimethyl-1,4,6-triphenyl-	344250-05-1	C25H21NO	91307132	29.9	0.44	2.16

RT	Compound Name	CAS#	Formula	Area	Match Score	Sample	Sample
0.4861	Coronene, methyl-	13119-86-3	C25H14	1365892813	54.2	6.62	32.26

Coronene, methyl- (NIST17.L)

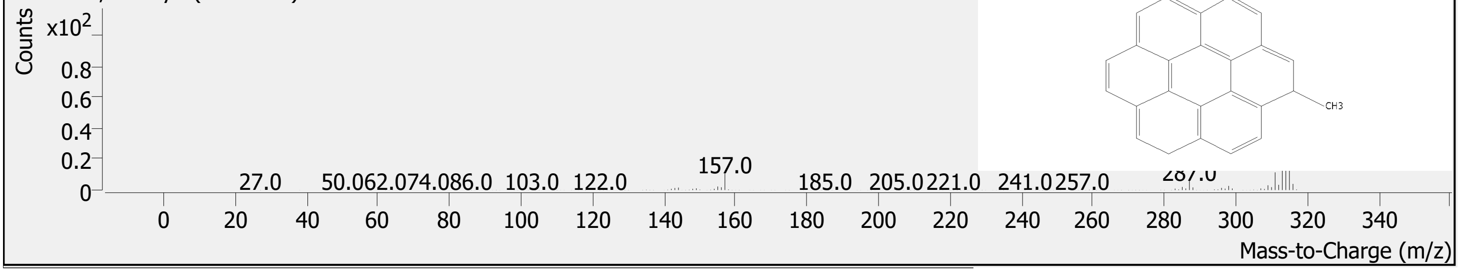

RT	Compound Name	CAS#	Formula	Area	Match Score	Sample	Sample
0.4861	11-Chlorodibenzo(a,c)phenazine	4618-89-7	C20H11ClN2	1365892813	54.0	6.62	32.26

11-Chlorodibenzo(a,c)phenazine (NIST17.L)

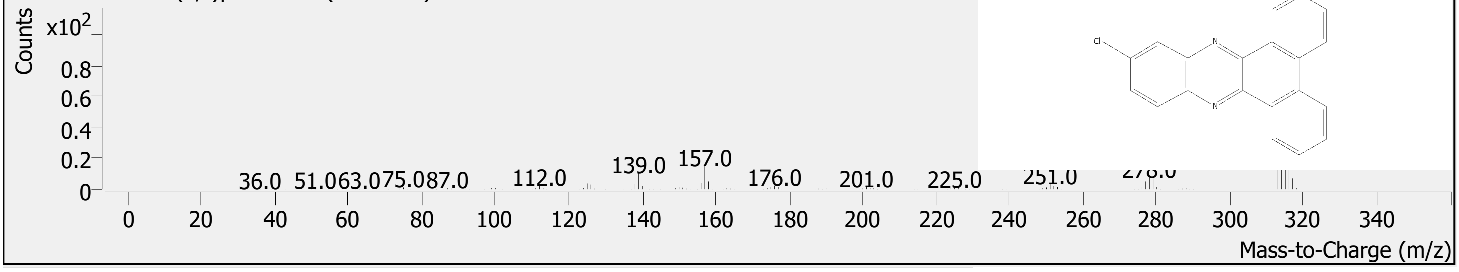

RT	Compound Name	CAS#	Formula	Area	Match Score	Sample	Sample
0.4861	4-Chloro-2-{9-fluoro-[1,2,4]triazolo[1,5-c]quinazolin-2-yl}phenol	1000435-69-4	C15H8ClFN4O	1365892813	51.7	6.62	32.26

4-Chloro-2-{9-fluoro-[1,2,4]triazolo[1,5-c]quinazolin-2-yl}phenol (NIST17.L)

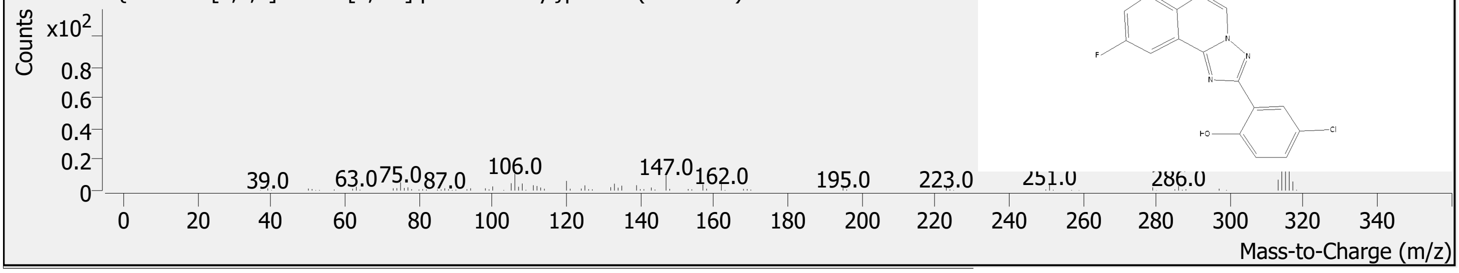

RT	Compound Name	CAS#	Formula	Area	Match Score	Sample	Sample
1.1206	2-Phenylethyl docosanoate	104899-74-3	C30H52O2	561659522	69.0	2.72	13.26

2-Phenylethyl docosanoate (NIST17.L)

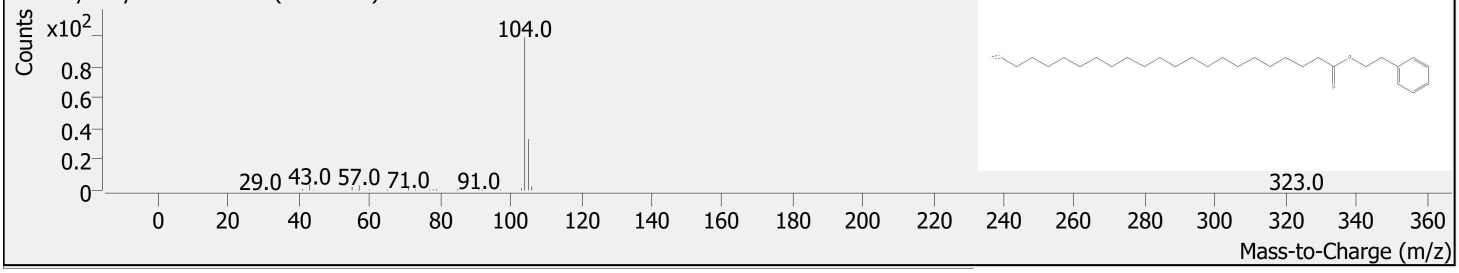

RT	Compound Name	CAS#	Formula	Area	Match Score	Sample	Sample
1.1206	Succinic acid, hexyl 2-phenylethyl ester	1000325-03-7	C18H26O4	561659522	67.9	2.72	13.26

Succinic acid, hexyl 2-phenylethyl ester (NIST17.L)

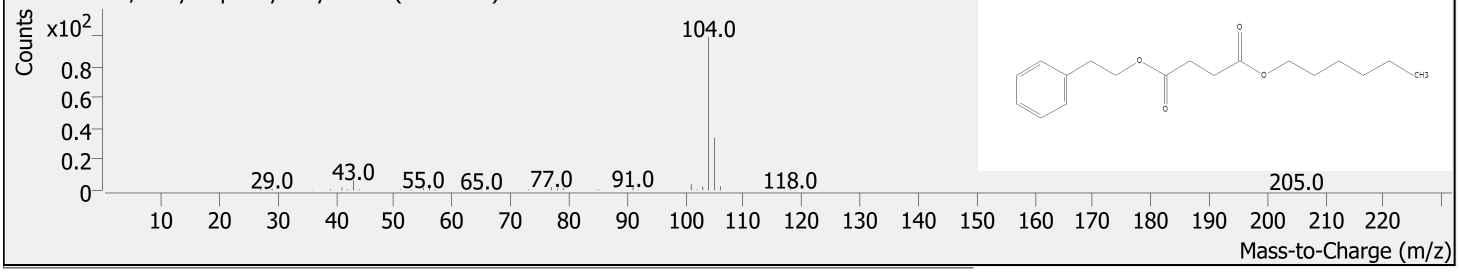

RT	Compound Name	CAS#	Formula	Area	Match Score	Sample	Sample
1.1206	Succinic acid, octyl 2-phenylethyl ester	1000325-03-9	C20H30O4	561659522	67.1	2.72	13.26

Succinic acid, octyl 2-phenylethyl ester (NIST17.L)

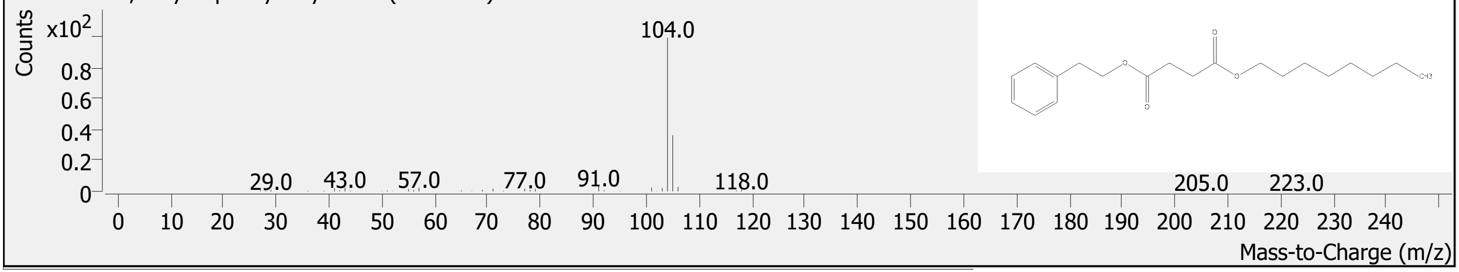

RT	Compound Name	CAS#	Formula	Area	Match Score	Sample	Sample
1.2277	Zirconium, dichloro-[dimethylbis(2-methyl-4-phenylindenyl)silane]	1000164-77-3	C34H30Cl2Si Zr	300295597	57.8	1.46	7.09

Zirconium, dichloro-[dimethylbis(2-methyl-4-phenylindenyl)silane] (NIST17.L)

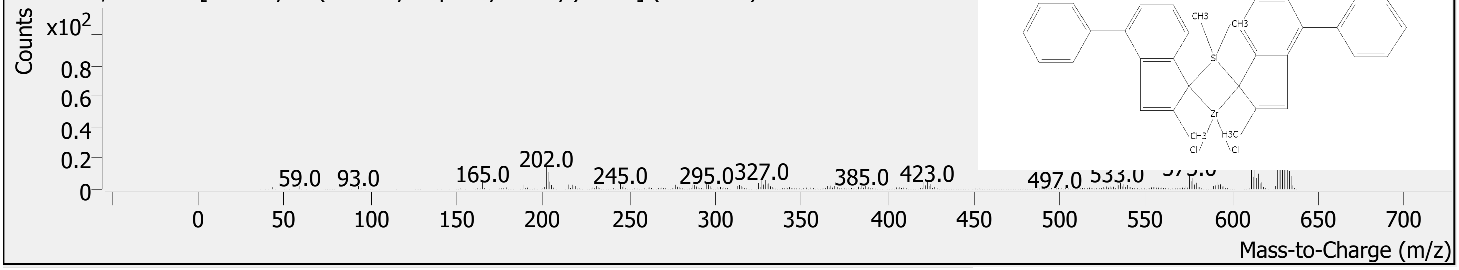

RT	Compound Name	CAS#	Formula	Area	Match Score	Sample	Sample
1.2277	2,2':3',2'':3'',2'''-Quaternaphthalene-1,1',1'',1''',4,4',4'',4'''-octone	125520-49-2	C40H18O8	300295597	42.7	1.46	7.09

2,2':3',2'':3'',2'''-Quaternaphthalene-1,1',1'',1''',4,4',4'',4'''-octone (NIST17.L)

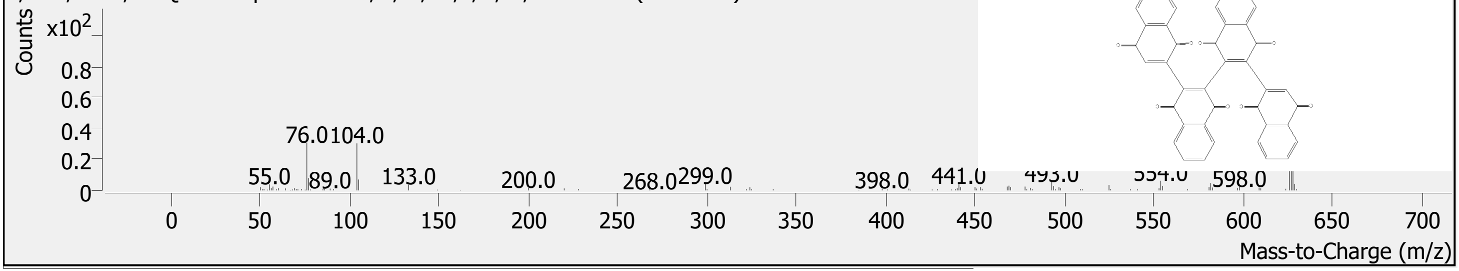

RT	Compound Name	CAS#	Formula	Area	Match Score	Sample	Sample
1.2277	benzenemethanamine, N,N'-(cyclohexylidenedi-4,1-phenylene)bis[N'-(phenylmethyl)-	1000402-99-8	C46H46N2	300295597	42.5	1.46	7.09

benzenemethanamine, N,N'-(cyclohexylidenedi-4,1-phenylene)bis[N'-(phenylmethyl)- (NIST17.L)

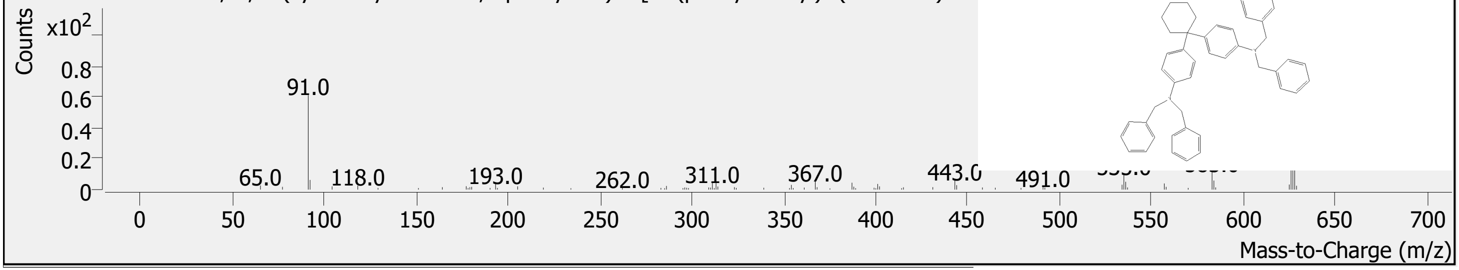

RT	Compound Name	CAS#	Formula	Area	Match Score	Sample	Sample
1.3348	2-Amino-3-(3,4-dimethoxyphenyl)-4H-benzo[g]chromen-4-one ditms	1000331-93-2	C27H33NO4 Si2	309577549	46.3	1.50	7.31

2-Amino-3-(3,4-dimethoxyphenyl)-4H-benzo[g]chromen-4-one ditms (NIST17.L)

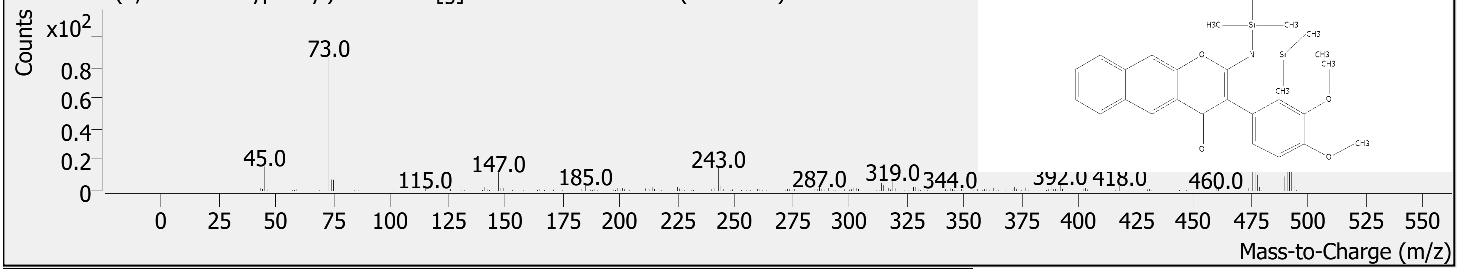

RT	Compound Name	CAS#	Formula	Area	Match Score	Sample	Sample
1.3348	1,4-Bis(2-naphthyloxy)anthraquinone	1000110-90-5	C34H20O4	309577549	45.3	1.50	7.31

1,4-Bis(2-naphthyloxy)anthraquinone (NIST17.L)

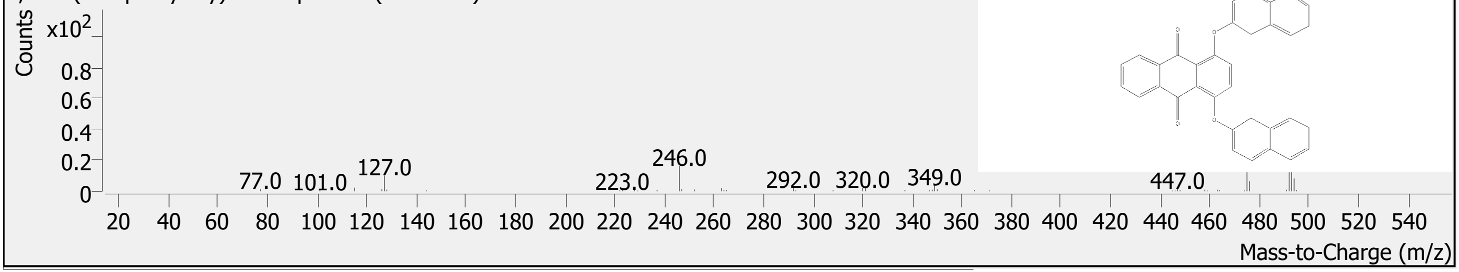

RT	Compound Name	CAS#	Formula	Area	Match Score	Sample	Sample
1.3348	2,8,10,12,18-Pentamethyl-3,7,13,17-tetraethyl-21H,23H-porphine	105043-42-3	C33H40N4	309577549	43.8	1.50	7.31

2,8,10,12,18-Pentamethyl-3,7,13,17-tetraethyl-21H,23H-porphine (NIST17.L)

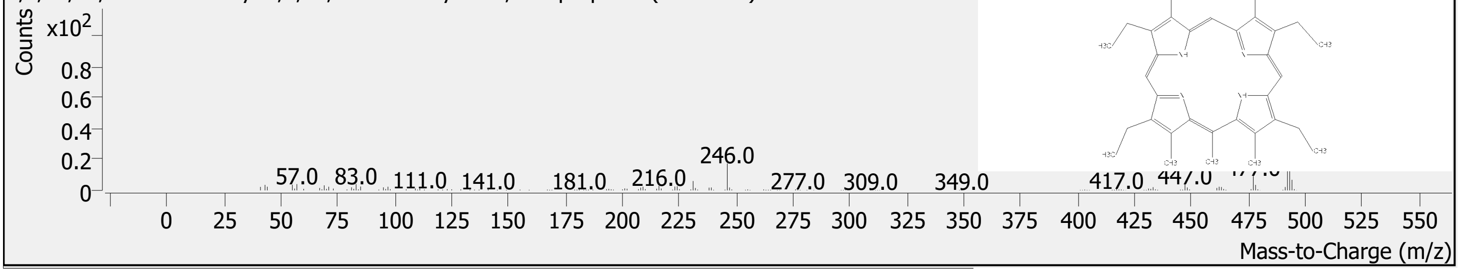

RT	Compound Name	CAS#	Formula	Area	Match Score	Sample	Sample
1.4337	2-Phenylethyl docosanoate	104899-74-3	C30H52O2	289891823	62.8	1.40	6.85

2-Phenylethyl docosanoate (NIST17.L)

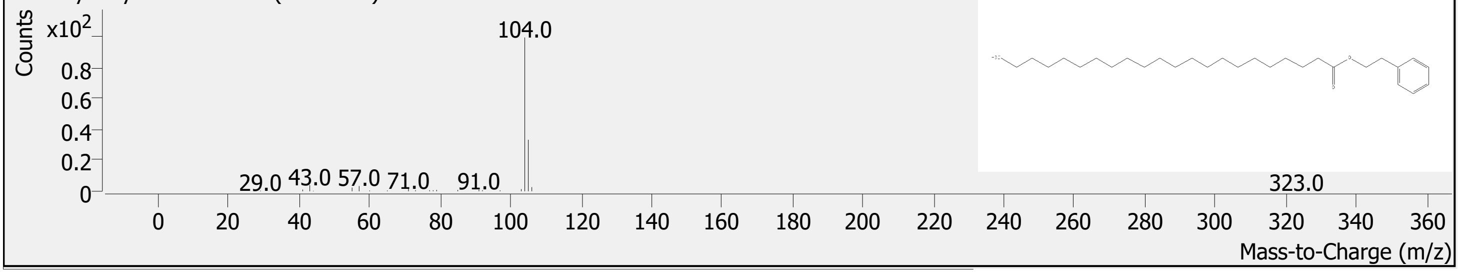

RT	Compound Name	CAS#	Formula	Area	Match Score	Sample	Sample
1.4337	Adipic acid, di(2-phenylethyl) ester	1000324-58-0	C22H26O4	289891823	62.1	1.40	6.85

Adipic acid, di(2-phenylethyl) ester (NIST17.L)

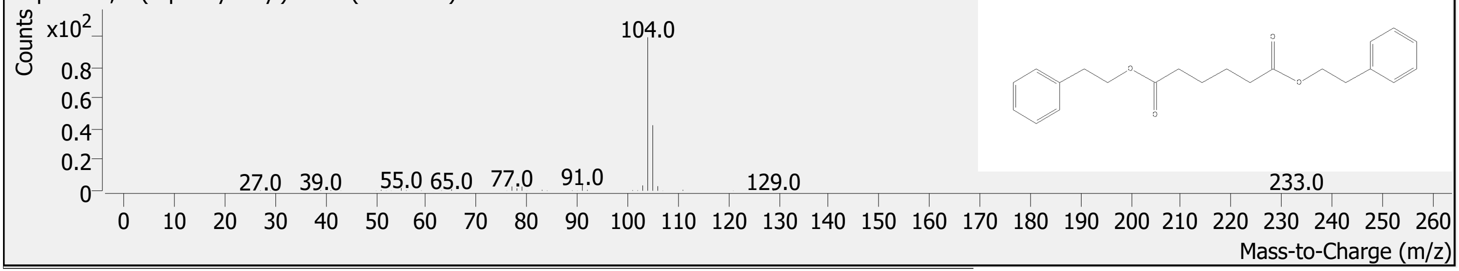

RT	Compound Name	CAS#	Formula	Area	Match Score	Sample	Sample
1.4337	Pimelic acid, di(phenethyl) ester	1000416-50-6	C23H28O4	289891823	61.9	1.40	6.85

Pimelic acid, di(phenethyl) ester (NIST17.L)

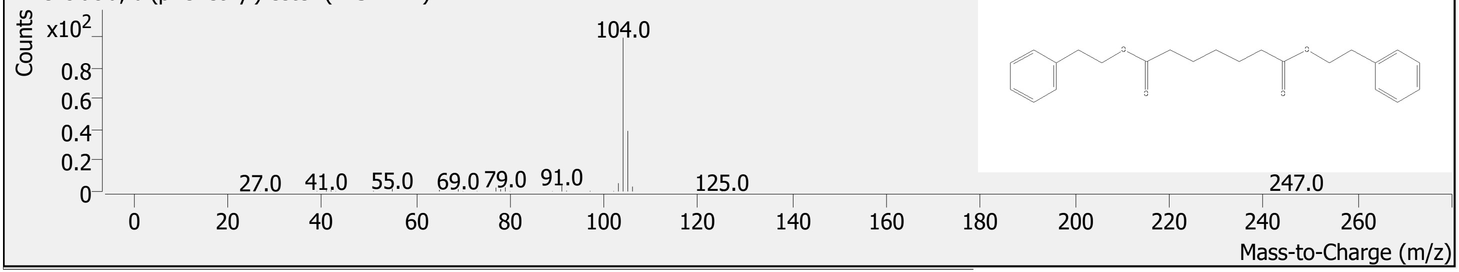

RT	Compound Name	CAS#	Formula	Area	Match Score	Sample	Sample
1.5573	2-(2-Fluoro-6a,8a,10,10-tetramethyl-4-oxo-7-(2,2,2-trifluoroacetoxy)-2,4,5,6,6a,6b,7,8,8a,8b,11a,12,12a,12b-tetradecahydro-1H-naphtho[2',1':4,5]indeno[1,2-d][1,3]dioxol-8b-yl)-2-oxoethyl 2,2,2-trifluoroacetate	1000385-76-0	C28H31F7O8	720017745	42.7	3.49	17.00

2-(2-Fluoro-6a,8a,10,10-tetramethyl-4-oxo-7-(2,2,2-trifluoroacetoxy)-2,4,5,6,6a,6b,7,8,8a,8b,11a,12

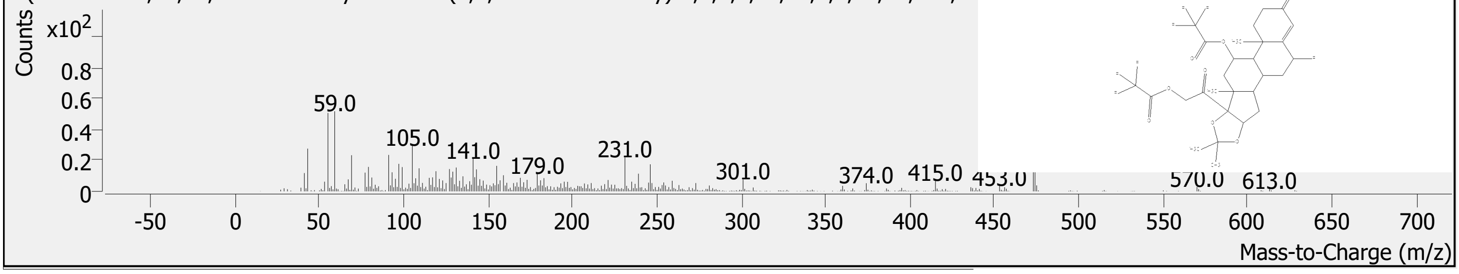

RT	Compound Name	CAS#	Formula	Area	Match Score	Sample	Sample
1.5573	(S)-7-Bromo-3-isobutyl-2-(2-methylbenzyl)-3,4-dihydro-2H-benzo[b][1,4,5]oxathiazepine 1,1-dioxide	1233355-16-2	C20H24BrNO3S	720017745	42.2	3.49	17.00

(S)-7-Bromo-3-isobutyl-2-(2-methylbenzyl)-3,4-dihydro-2H-benzo[b][1,4,5]oxathiazepine 1,1-dioxide

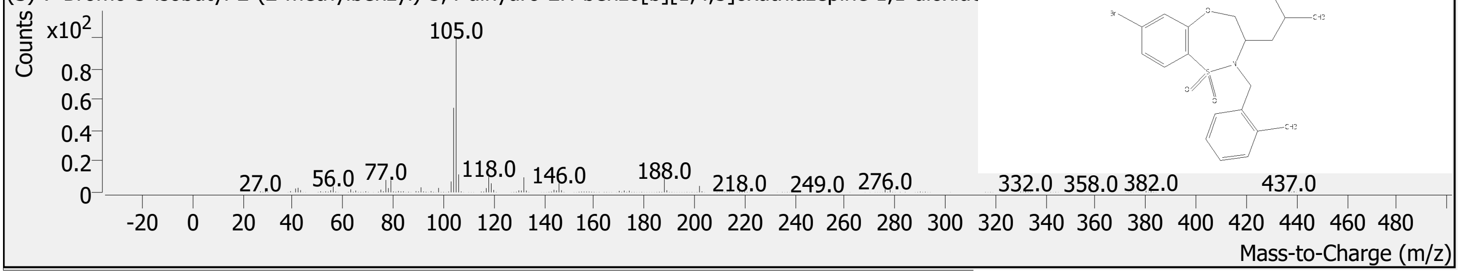

RT	Compound Name	CAS#	Formula	Area	Match Score	Sample	Sample
1.5573	Sebacic acid, 2-methylbenzyl undecyl ester	1000380-72-1	C29H48O4	720017745	39.2	3.49	17.00

Sebacic acid, 2-methylbenzyl undecyl ester (NIST17.L)

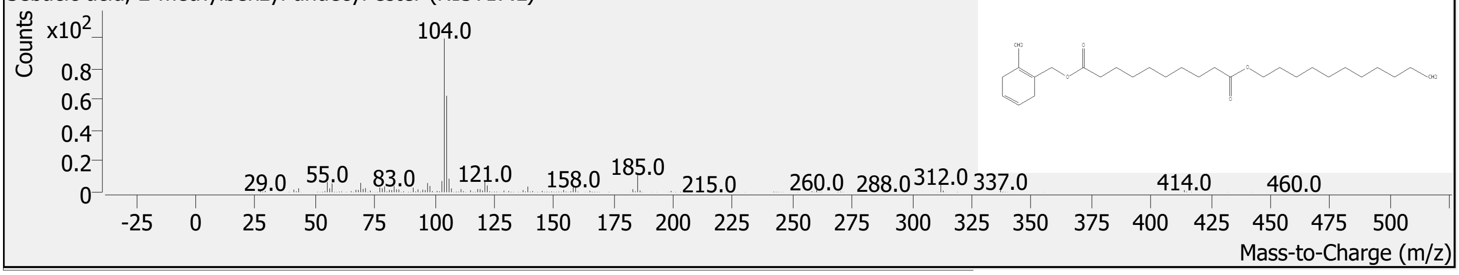

RT	Compound Name	CAS#	Formula	Area	Match Score	Sample	Sample
2.6450	4-(2-Chloro-phenyl)-1-cyclopropyl-1,4-dihydro-pyridine-3,5-dicarboxylic acid dimethyl ester	1000275-22-1	C18H18ClNO4	241504509	49.5	1.17	5.70

4-(2-Chloro-phenyl)-1-cyclopropyl-1,4-dihydro-pyridine-3,5-dicarboxylic acid dimethyl ester (NIST17.

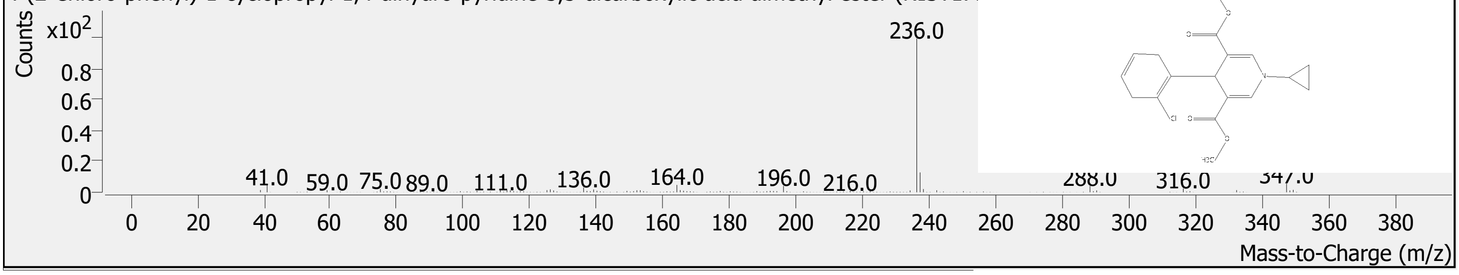

RT	Compound Name	CAS#	Formula	Area	Match Score	Sample	Sample
2.6450	4-(6-Chloro-benzo[1,3]dioxol-5-yl)-1-cyclopropyl-1,4-dihydro-pyridine-3,5-dicarboxylic acid dimethyl ester	1000275-03-3	C19H18ClNO6	241504509	48.6	1.17	5.70

4-(6-Chloro-benzo[1,3]dioxol-5-yl)-1-cyclopropyl-1,4-dihydro-pyridine-3,5-dicarboxylic acid dimethyl

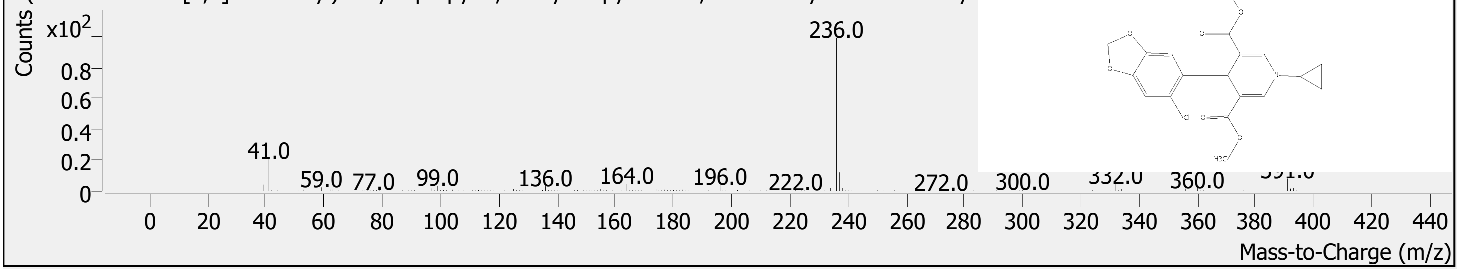

RT	Compound Name	CAS#	Formula	Area	Match Score	Sample	Sample
2.6450	1-Cyclopropyl-4-(4-methoxycarbonyl-phenyl)-1,4-dihydro-pyridine-3,5-dicarboxylic acid dimethyl ester	1000275-22-2	C20H21NO6	241504509	45.5	1.17	5.70

1-Cyclopropyl-4-(4-methoxycarbonyl-phenyl)-1,4-dihydro-pyridine-3,5-dicarboxylic acid dimethyl este

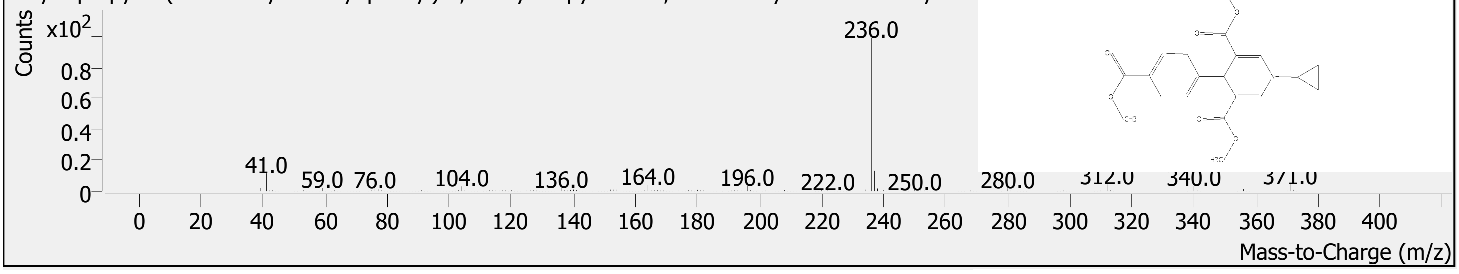

RT	Compound Name	CAS#	Formula	Area	Match Score	Sample	Sample
11.7256	N-(5,6-Dichloro-1,3-benzothiazol-2-yl)-2,2,2-trifluoroacetamide	1000373-29-0	C9H3Cl2F3N2OS	3331528237	42.3	16.15	78.67

N-(5,6-Dichloro-1,3-benzothiazol-2-yl)-2,2,2-trifluoroacetamide (NIST17.L)

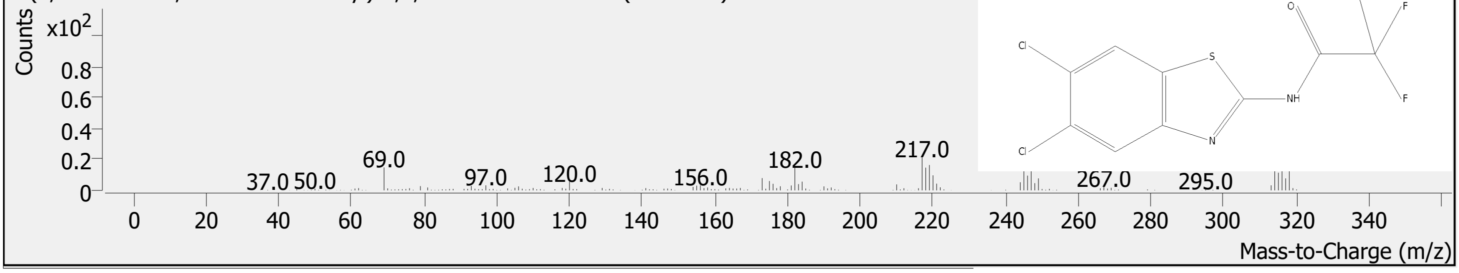

RT	Compound Name	CAS#	Formula	Area	Match Score	Sample	Sample
11.7256	(1H)Benzimidazole, 5-fluoro-2-(2-thienyl)-6-(4-methylpiperazin-1-yl)-	174468-69-0	C16H17FN4S	3331528237	38.3	16.15	78.67

(1H)Benzimidazole, 5-fluoro-2-(2-thienyl)-6-(4-methylpiperazin-1-yl)- (NIST17.L)

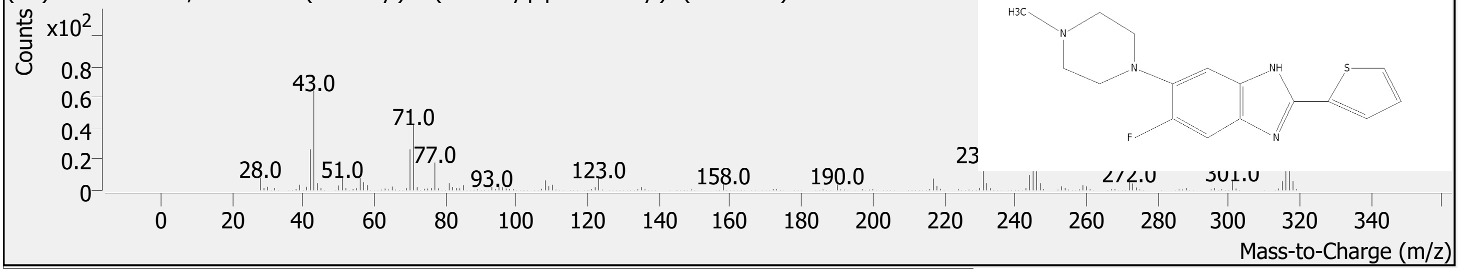

RT	Compound Name	CAS#	Formula	Area	Match Score	Sample	Sample
11.7256	6-Bromo-2-methoxy-4-phenyl-quinazoline	1000318-41-9	C15H11BrN2O	3331528237	37.5	16.15	78.67

6-Bromo-2-methoxy-4-phenyl-quinazoline (NIST17.L)

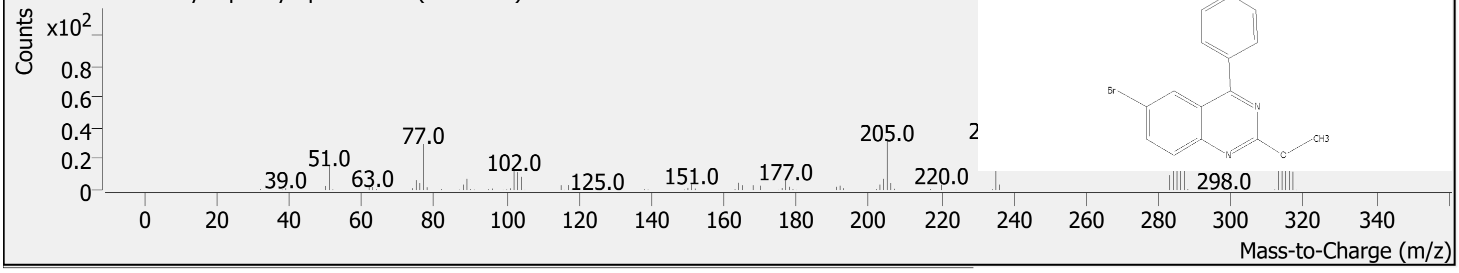

RT	Compound Name	CAS#	Formula	Area	Match Score	Sample	Sample
12.4590	11-Chlorodibenzo(a,c)phenazine	4618-89-7	C20H11ClN2	2222681794	43.2	10.77	52.49

11-Chlorodibenzo(a,c)phenazine (NIST17.L)

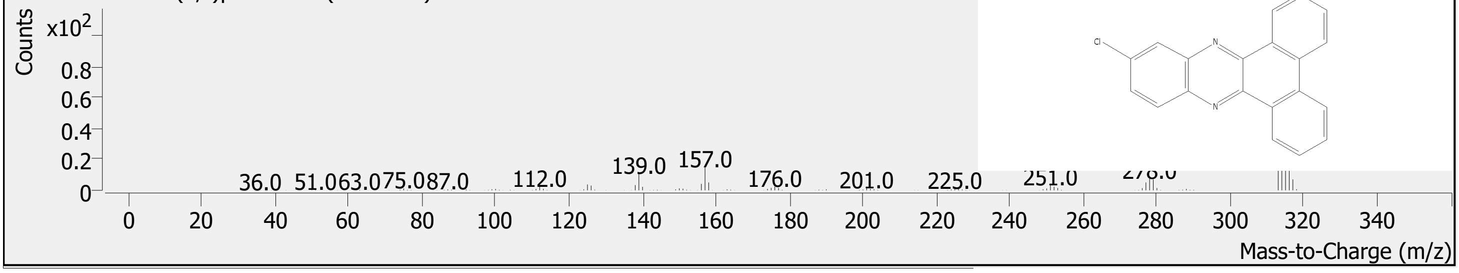

RT	Compound Name	CAS#	Formula	Area	Match Score	Sample	Sample
12.4590	Coronene, methyl-	13119-86-3	C25H14	2222681794	41.1	10.77	52.49

Coronene, methyl- (NIST17.L)

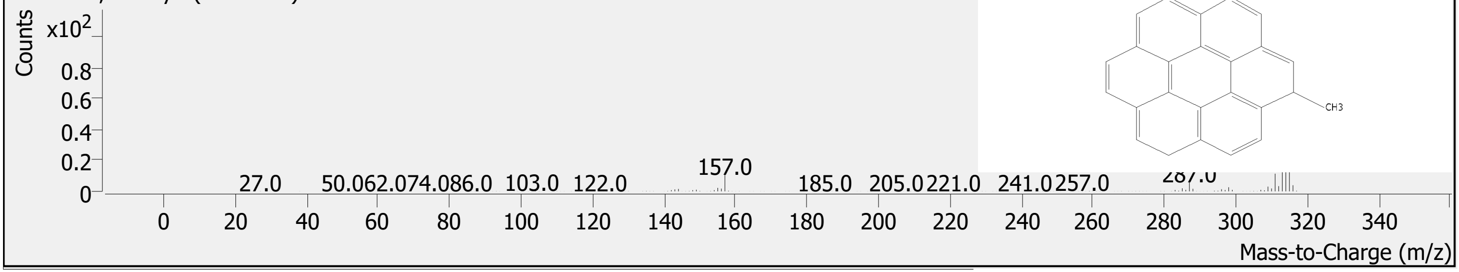

RT	Compound Name	CAS#	Formula	Area	Match Score	Sample	Sample
12.4590	5'-Fluoro-2'-(tert.-butyldimethylsilyl)oxy-4-methylchalcone	1000454-17-9	C22H27FO2 Si	2222681794	40.7	10.77	52.49

5'-Fluoro-2'-(tert.-butyldimethylsilyl)oxy-4-methylchalcone (NIST17.L)

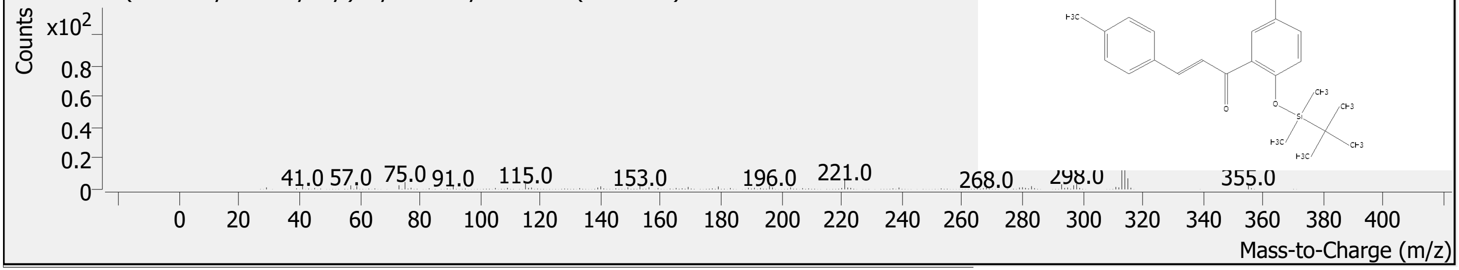

RT	Compound Name	CAS#	Formula	Area	Match Score	Sample	Sample
12.7144	Zirconium, dichloro-[dimethylbis(2-methyl-4-phenylindenyl)silane]	1000164-77-3	C34H30Cl2Si Zr	1502170002	55.5	7.28	35.47

Zirconium, dichloro-[dimethylbis(2-methyl-4-phenylindenyl)silane] (NIST17.L)

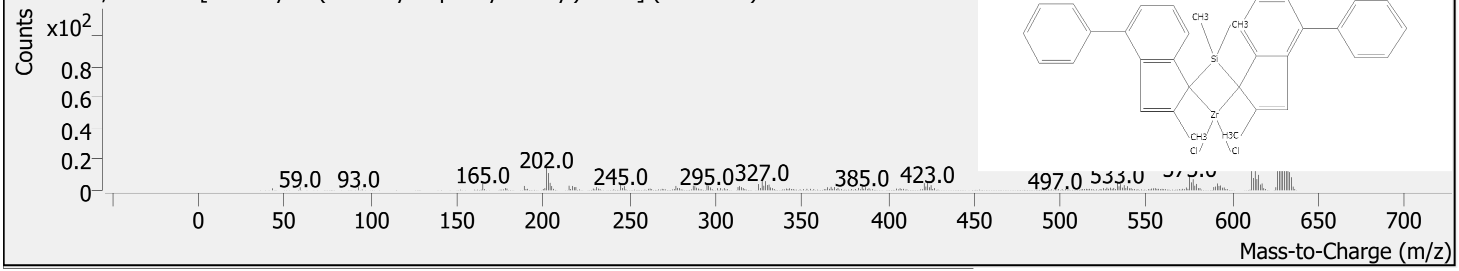

RT	Compound Name	CAS#	Formula	Area	Match Score	Sample	Sample
12.7144	Yohimban-16-carboxylic acid, 17-hydroxy-, methyl ester, (16.beta.,17.alpha.)-	483-10-3	C21H26N2O 3	1502170002	43.7	7.28	35.47

Yohimban-16-carboxylic acid, 17-hydroxy-, methyl ester, (16.beta.,17.alpha.)- (NIST17.L)

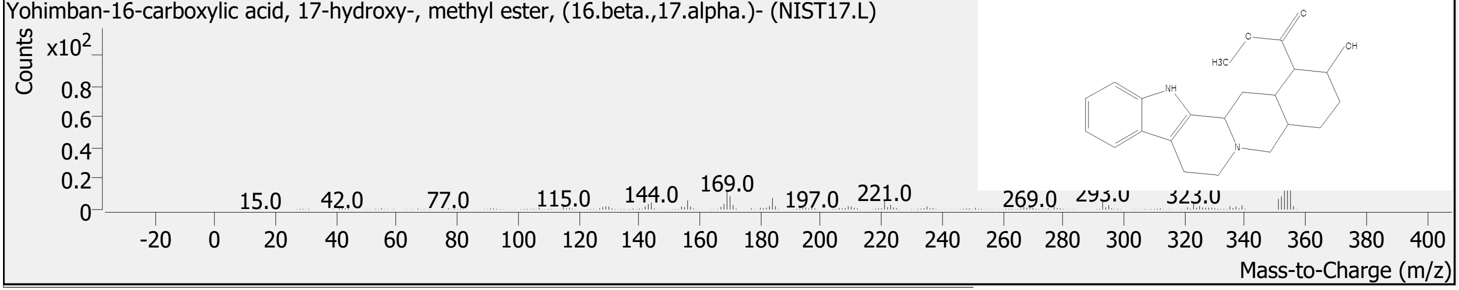

RT	Compound Name	CAS#	Formula	Area	Match Score	Sample	Sample
12.7144	Gallacetophenone-4'-methylether, bis(tert-butyldimethylsilyl) ether	1000462-95-3	C21H38O4Si 2	1502170002	43.2	7.28	35.47

Gallacetophenone-4'-methylether, bis(tert-butyldimethylsilyl) ether (NIST17.L)

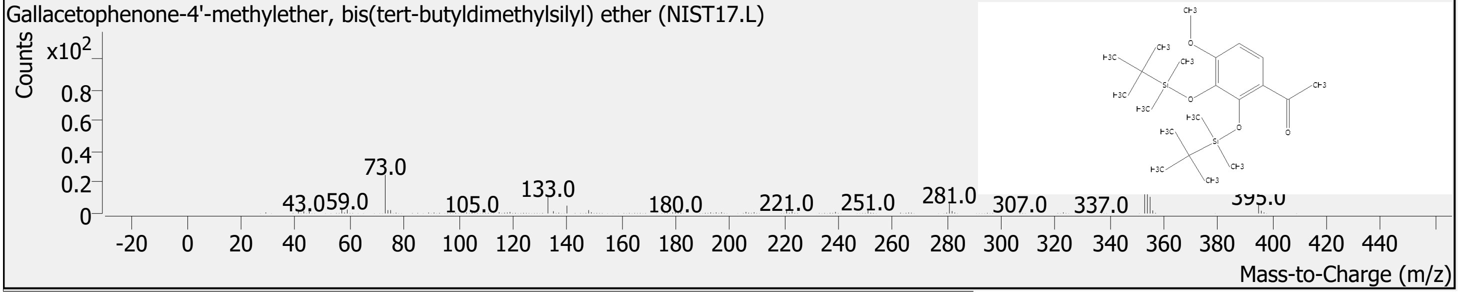

RT	Compound Name	CAS#	Formula	Area	Match Score	Sample	Sample
12.7556	11-Chlorodibenzo(a,c)phenazine	4618-89-7	C20H11ClN2	468472118	48.5	2.27	11.06

11-Chlorodibenzo(a,c)phenazine (NIST17.L)

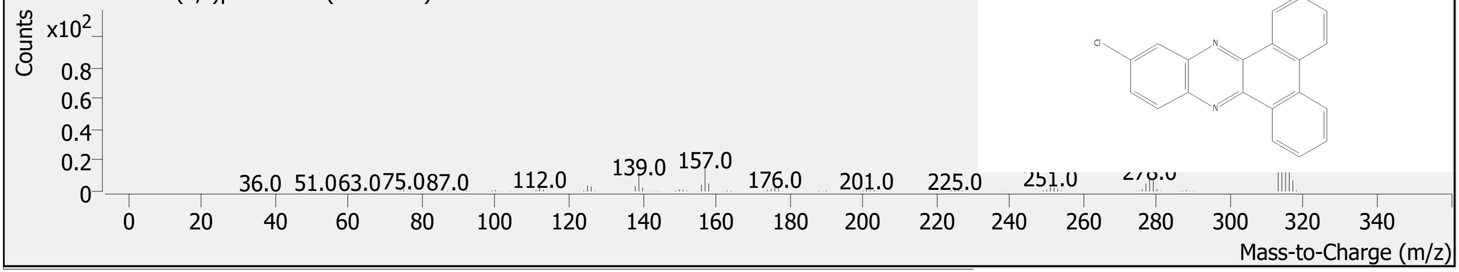

RT	Compound Name	CAS#	Formula	Area	Match Score	Sample	Sample
12.7556	Coronene, methyl-	13119-86-3	C25H14	468472118	48.1	2.27	11.06

Coronene, methyl- (NIST17.L)

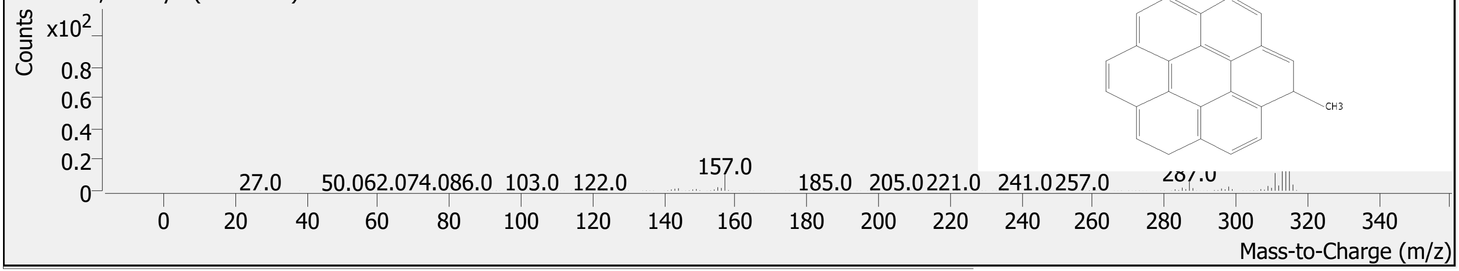

RT	Compound Name	CAS#	Formula	Area	Match Score	Sample	Sample
12.7556	7-Bromo-5-chloro-8-hydroxyquinoline, tert-butyldimethylsilyl ether	1000463-53-6	C15H19BrCl NOSi	468472118	46.8	2.27	11.06

7-Bromo-5-chloro-8-hydroxyquinoline, tert-butyldimethylsilyl ether (NIST17.L)

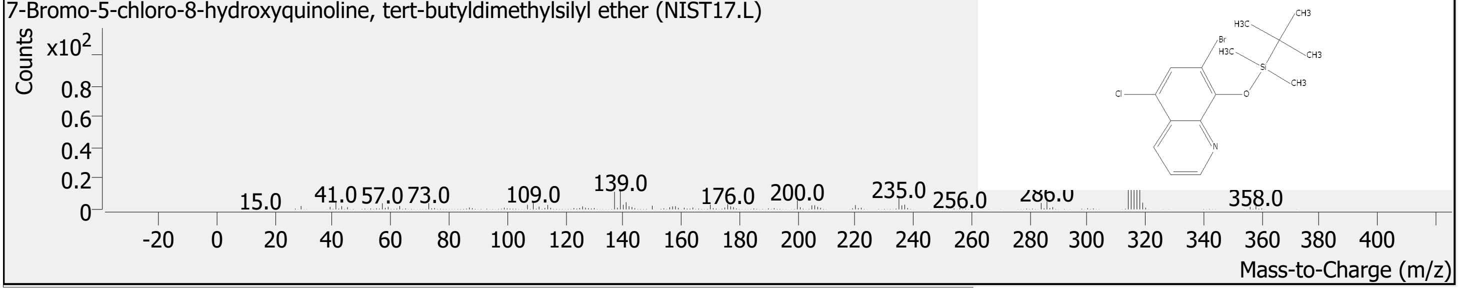

RT	Compound Name	CAS#	Formula	Area	Match Score	Sample	Sample
12.8627	Coronene, methyl-	13119-86-3	C25H14	520718585	55.1	2.52	12.30

Coronene, methyl- (NIST17.L)

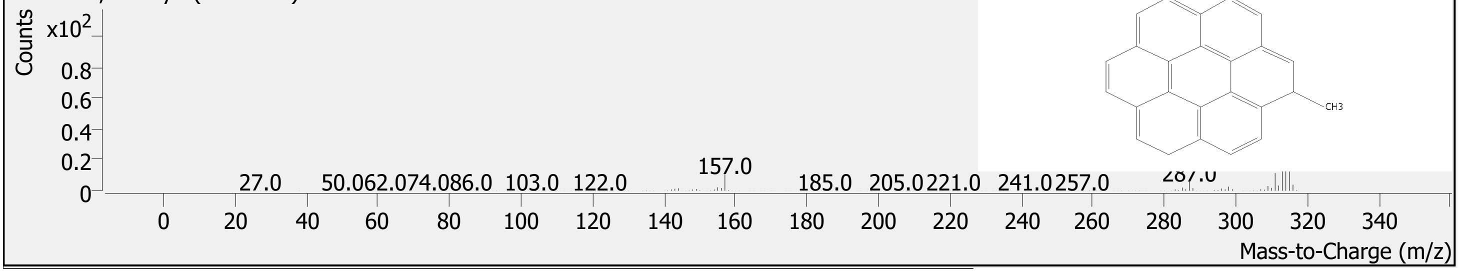

RT	Compound Name	CAS#	Formula	Area	Match Score	Sample	Sample
12.8627	5'-Fluoro-2'-(tert.-butyldimethylsilyl)oxy-4-methylchalcone	1000454-17-9	C22H27FO2 Si	520718585	52.4	2.52	12.30

5'-Fluoro-2'-(tert.-butyldimethylsilyl)oxy-4-methylchalcone (NIST17.L)

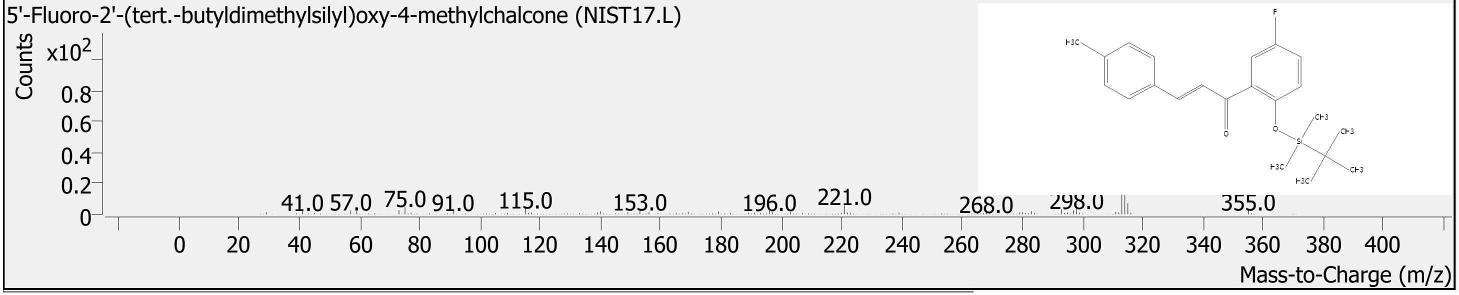

RT	Compound Name	CAS#	Formula	Area	Match Score	Sample	Sample
12.8627	11-Chlorodibenzo(a,c)phenazine	4618-89-7	C20H11ClN2	520718585	51.5	2.52	12.30

Agilent Trusted Answers

11-Chlorodibenzo(a,c)phenazine (NIST17.L)

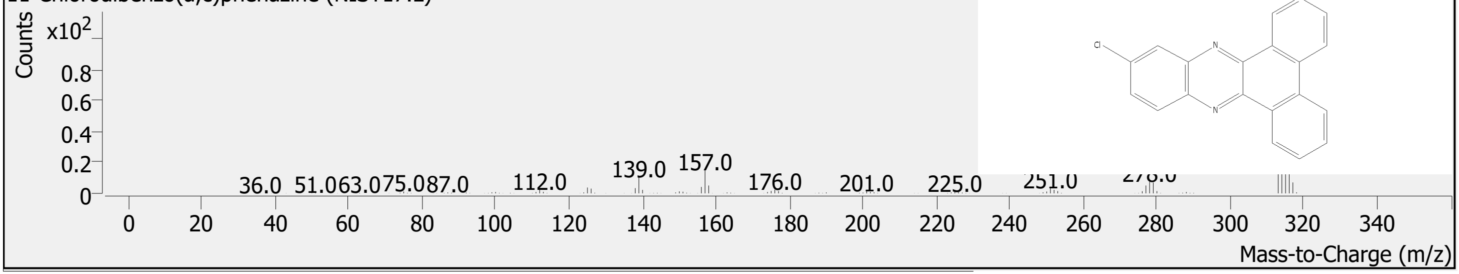

RT	Compound Name	CAS#	Formula	Area	Match Score	Sample	Sample
12.9369	9,9'-Spirobifluorene	1000326-43-1	C25H16	1076500291	43.5	5.22	25.42

9,9'-Spirobifluorene (NIST17.L)

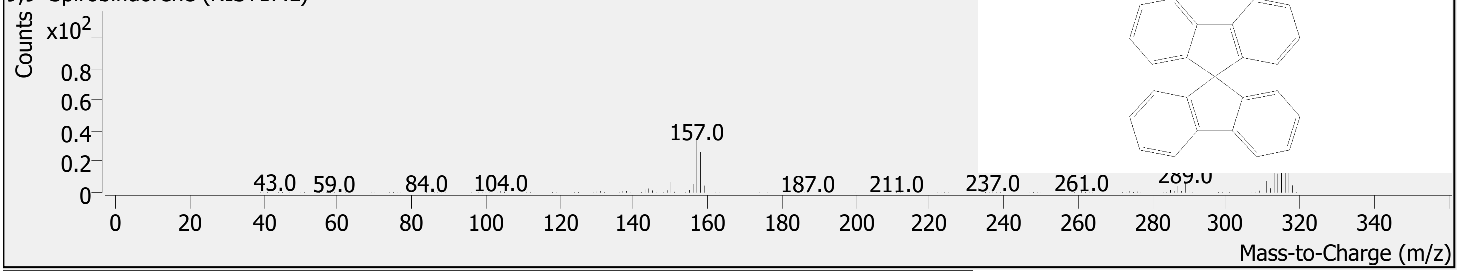

RT	Compound Name	CAS#	Formula	Area	Match Score	Sample	Sample
12.9369	6H-Benzo[b]naphtho[2,3-h]carbazole	905-95-3	C24H15N	1076500291	42.7	5.22	25.42

6H-Benzo[b]naphtho[2,3-h]carbazole (NIST17.L)

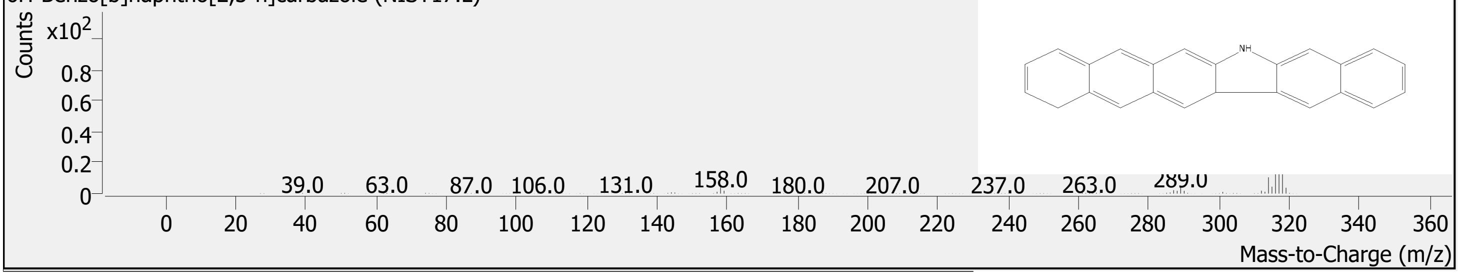

RT	Compound Name	CAS#	Formula	Area	Match Score	Sample	Sample
12.9369	15H-Benzo[a]naphtho[2,3-h]carbazole	17182-04-6	C24H15N	1076500291	41.7	5.22	25.42

15H-Benzo[a]naphtho[2,3-h]carbazole (NIST17.L)

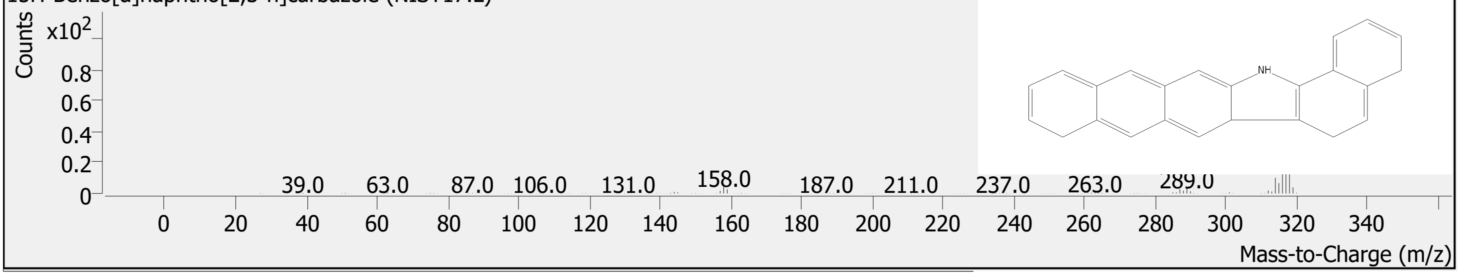

RT	Compound Name	CAS#	Formula	Area	Match Score	Sample	Sample
13.0358	11-Chlorodibenzo(a,c)phenazine	4618-89-7	C20H11ClN2	2544429052	49.0	12.33	60.09

11-Chlorodibenzo(a,c)phenazine (NIST17.L)

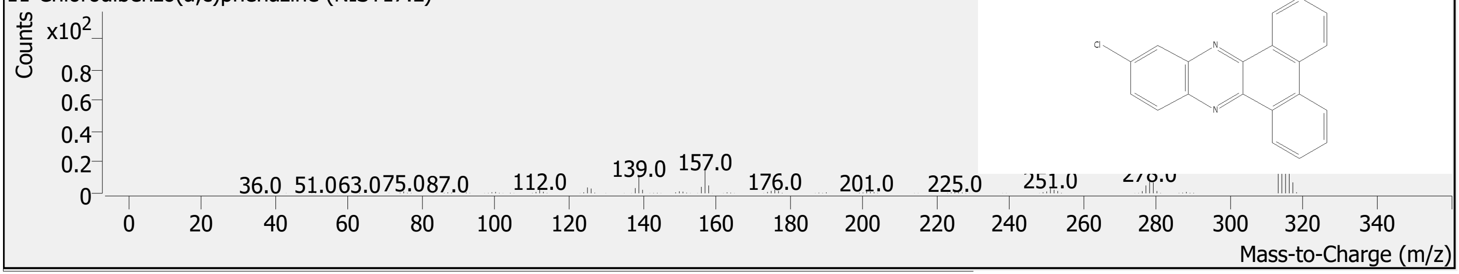

RT	Compound Name	CAS#	Formula	Area	Match Score	Sample	Sample
13.0358	7-Bromo-5-chloro-8-hydroxyquinoline, tert-butyldimethylsilyl ether	1000463-53-6	C15H19BrCl NOSi	2544429052	46.5	12.33	60.09

7-Bromo-5-chloro-8-hydroxyquinoline, tert-butyldimethylsilyl ether (NIST17.L)

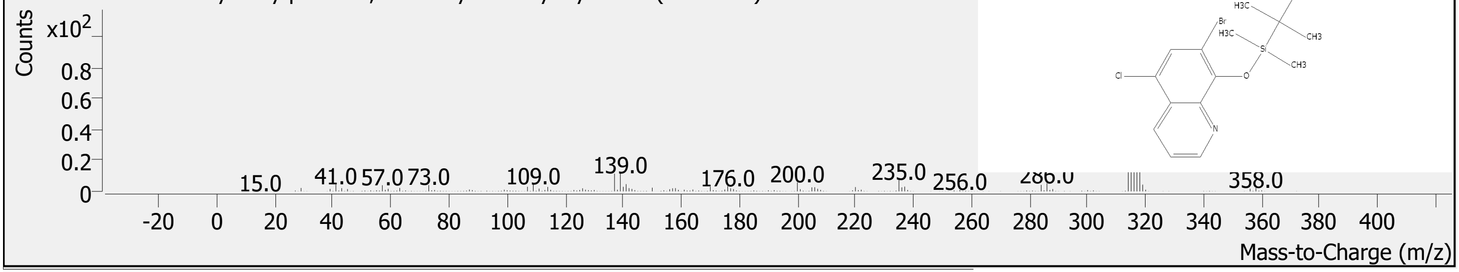

RT	Compound Name	CAS#	Formula	Area	Match Score	Sample	Sample
13.0358	9,9'-Spirobifluorene	1000326-43-1	C25H16	2544429052	46.3	12.33	60.09

9,9'-Spirobifluorene (NIST17.L)

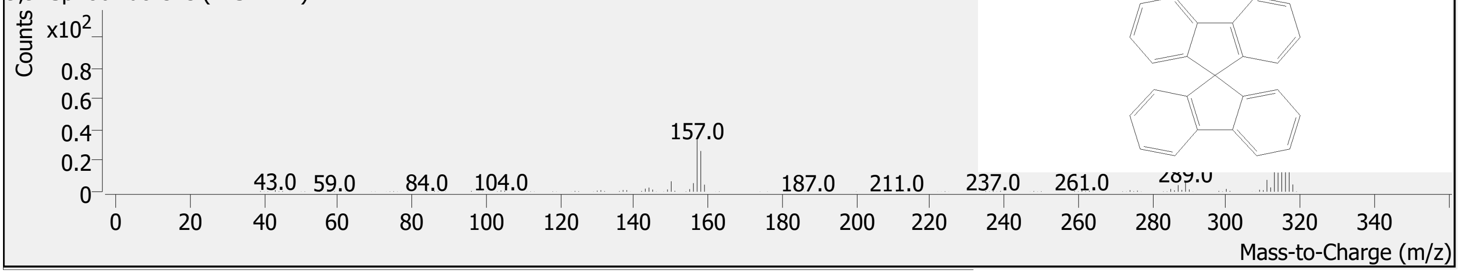

RT	Compound Name	CAS#	Formula	Area	Match Score	Sample	Sample
13.3324	5'-Fluoro-2'-(tert.-butyldimethylsilyl)oxy-4-methylchalcone	1000454-17-9	C22H27FO2 Si	1654231499	46.2	8.02	39.06

5'-Fluoro-2'-(tert.-butyldimethylsilyl)oxy-4-methylchalcone (NIST17.L)

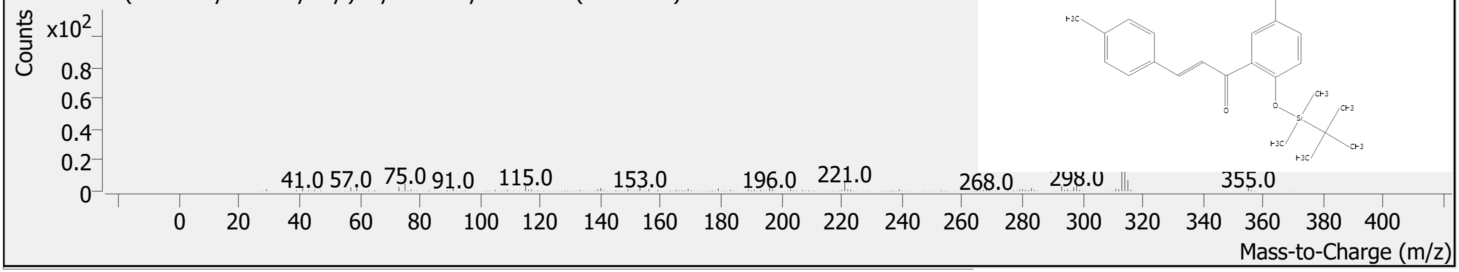

RT	Compound Name	CAS#	Formula	Area	Match Score	Sample	Sample
13.3324	Estra-1,3,5(10)-trien-17-one, 3-(acetyloxy)-4-nitro-, 17-(O-methyloxime)	77883-07-9	C21H26N2O 5	1654231499	44.7	8.02	39.06

Estra-1,3,5(10)-trien-17-one, 3-(acetyloxy)-4-nitro-, 17-(O-methyloxime) (NIST17.L)

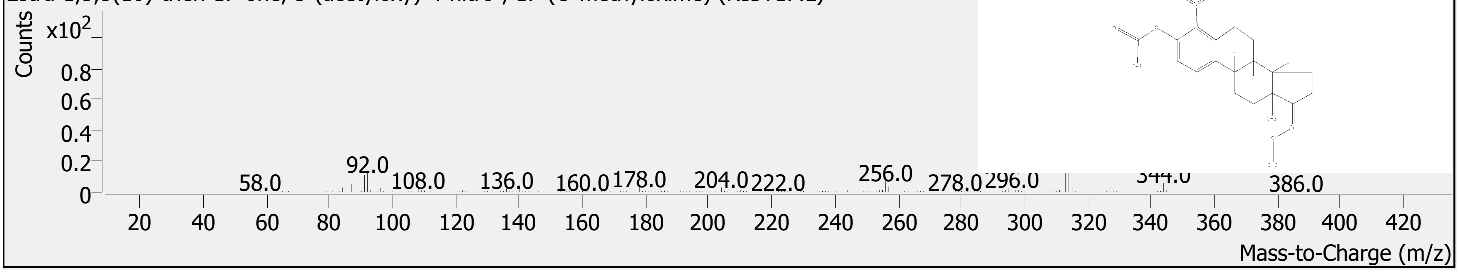

RT	Compound Name	CAS#	Formula	Area	Match Score	Sample	Sample
13.3324	Tungsten, [(1,2,3-.eta.)-2-methyl-2-propenyl]tris(.eta.3-2-propenyl)-	127629-47-4	C13H22W	1654231499	43.8	8.02	39.06

Tungsten, [(1,2,3-.eta.)-2-methyl-2-propenyl]tris(.eta.3-2-propenyl)- (NIST17.L)

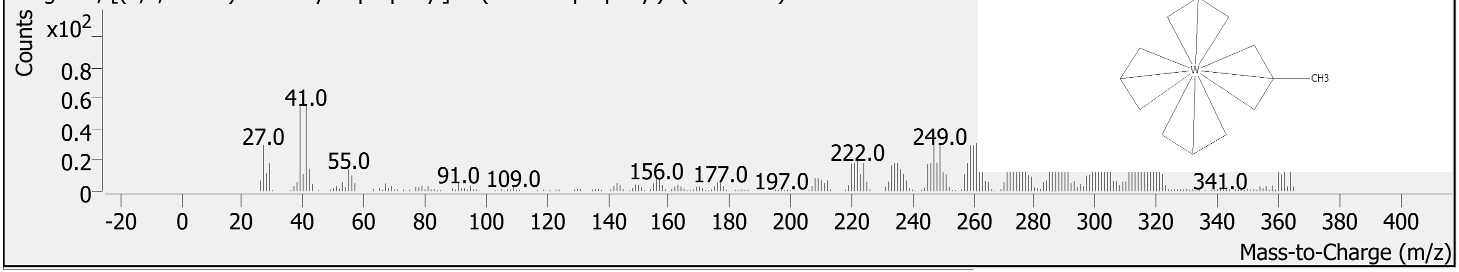

RT	Compound Name	CAS#	Formula	Area	Match Score	Sample	Sample
13.5631	Bis(.mu.-propynyl)-bis(cyclopentadienyl)zirconium-bis(t-butylcyclopentadienyl)zirconium	1000154-45-0	C34H42Zr2	1259765549	50.0	6.11	29.75

Bis(.mu.-propynyl)-bis(cyclopentadienyl)zirconium-bis(t-butylcyclopentadienyl)zirconium (NIST17.L)

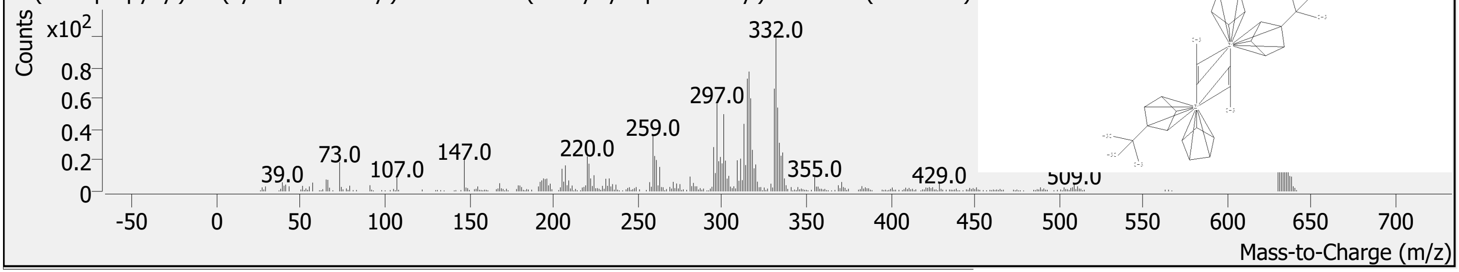

RT	Compound Name	CAS#	Formula	Area	Match Score	Sample	Sample
13.5631	5'-Fluoro-2'-(tert.-butyldimethylsilyl)oxy-4-methylchalcone	1000454-17-9	C22H27FO2Si	1259765549	46.1	6.11	29.75

5'-Fluoro-2'-(tert.-butyldimethylsilyl)oxy-4-methylchalcone (NIST17.L)

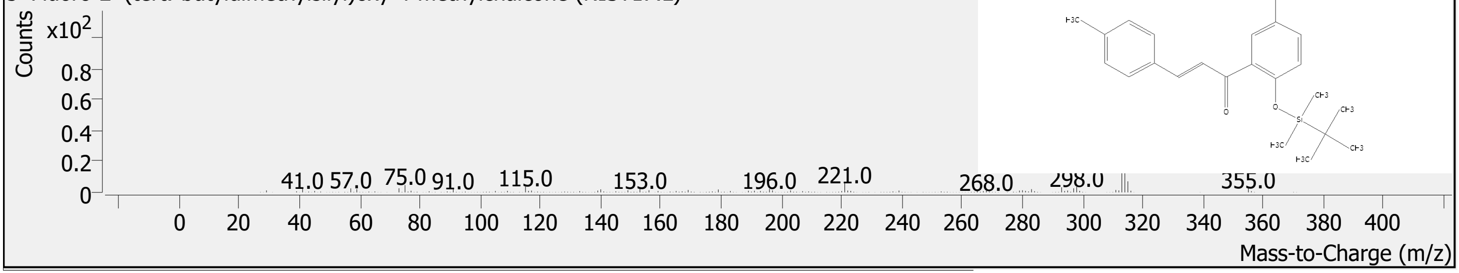

RT	Compound Name	CAS#	Formula	Area	Match Score	Sample	Sample
13.5631	1-(4-Hydroxyphenyl)-4-(4-chlorophenyl)-(1H)-pyrimidin-2-thione	1000286-95-8	C16H11ClN2OS	1259765549	43.0	6.11	29.75

1-(4-Hydroxyphenyl)-4-(4-chlorophenyl)-(1H)-pyrimidin-2-thione (NIST17.L)

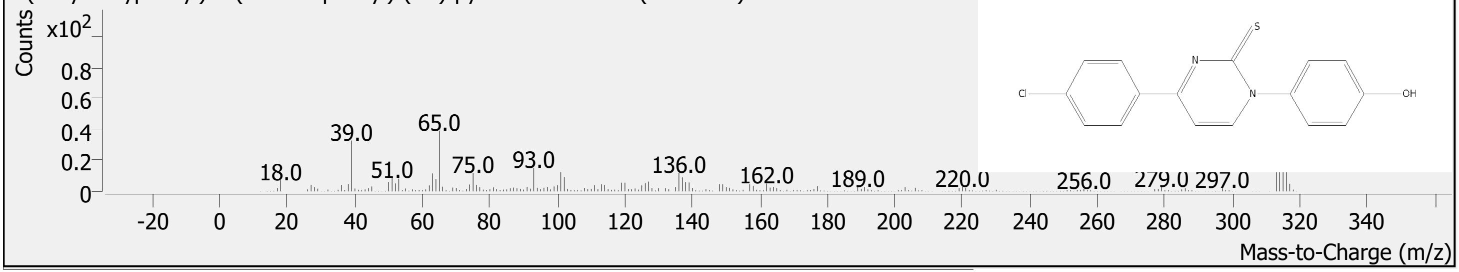

RT	Compound Name	CAS#	Formula	Area	Match Score	Sample	Sample
13.7032	7-Bromo-5-chloro-8-hydroxyquinoline, tert-butyldimethylsilyl ether	1000463-53-6	C15H19BrClNOSi	1864120693	44.7	9.03	44.02

7-Bromo-5-chloro-8-hydroxyquinoline, tert-butyldimethylsilyl ether (NIST17.L)

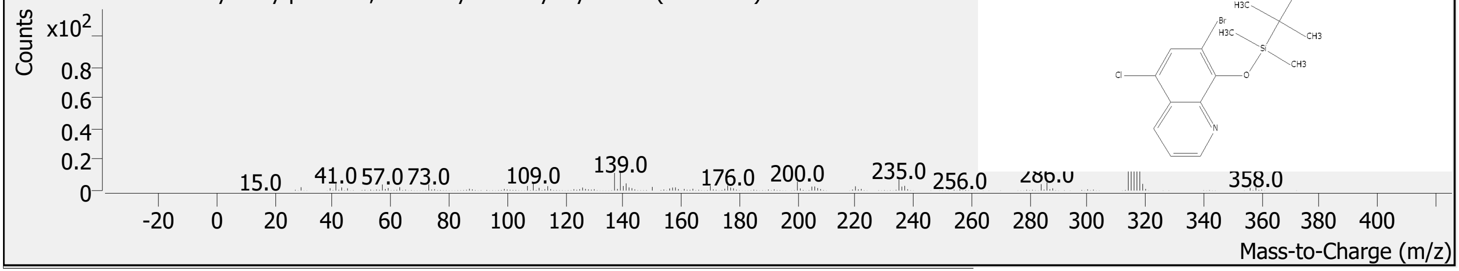

RT	Compound Name	CAS#	Formula	Area	Match Score	Sample	Sample
13.7032	11-Chlorodibenzo(a,c)phenazine	4618-89-7	C20H11ClN2	1864120693	43.2	9.03	44.02

11-Chlorodibenzo(a,c)phenazine (NIST17.L)

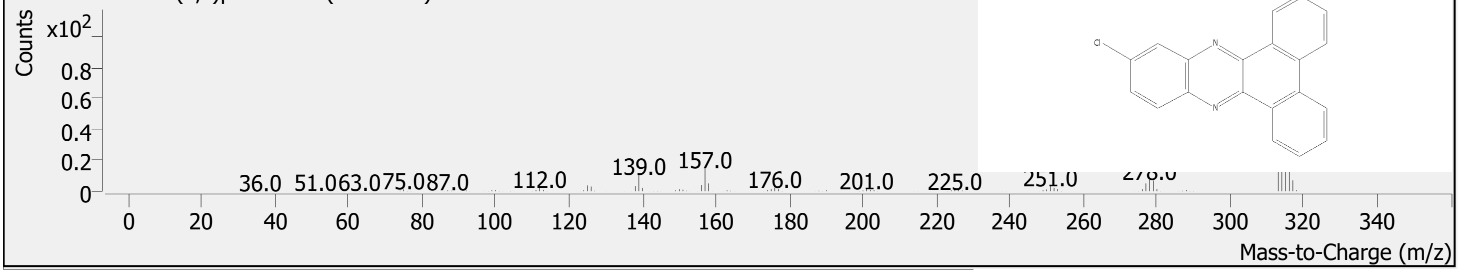

RT	Compound Name	CAS#	Formula	Area	Match Score	Sample	Sample
13.7032	7-Bromo-5-chloro-8-hydroxyquinoline, trimethylsilyl ether	1000463-53-9	C12H13BrClNOSi	1864120693	43.0	9.03	44.02

7-Bromo-5-chloro-8-hydroxyquinoline, trimethylsilyl ether (NIST17.L)

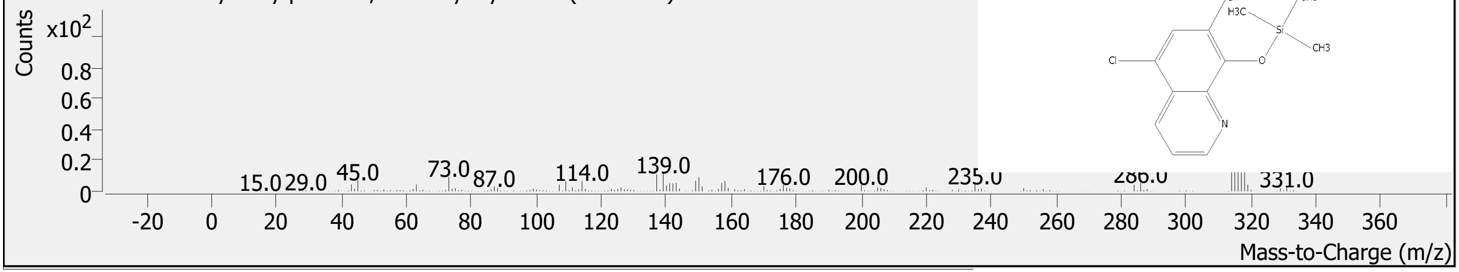

RT	Compound Name	CAS#	Formula	Area	Match Score	Sample	Sample
14.0493	Silane, diethylheptadecyloxy(2-methoxyethoxy)-	1000363-54-6	C24H52O3Si	4234649045	46.3	20.52	100.00

Silane, diethylheptadecyloxy(2-methoxyethoxy)- (NIST17.L)

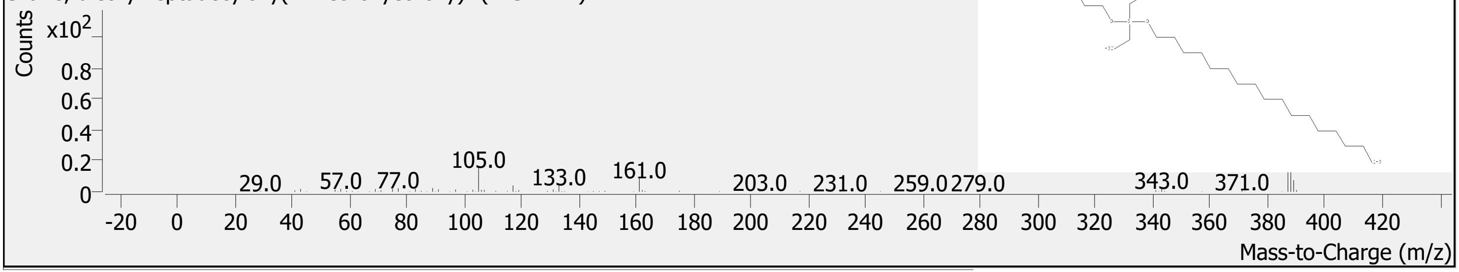

RT	Compound Name	CAS#	Formula	Area	Match Score	Sample	Sample
14.0493	acetic acid, 2,2'-[(2,2',3,3'-tetrahydro-3,3,3',3'-tetramethyl-1,1'-spirobi[1H-indene]-6,6'-diyl)bis(oxy)]bis-	1000399-17-5	C25H28O6	4234649045	44.8	20.52	100.00

acetic acid, 2,2'-[(2,2',3,3'-tetrahydro-3,3,3',3'-tetramethyl-1,1'-spirobi[1H-indene]-6,6'-diyl)bis(oxy)]

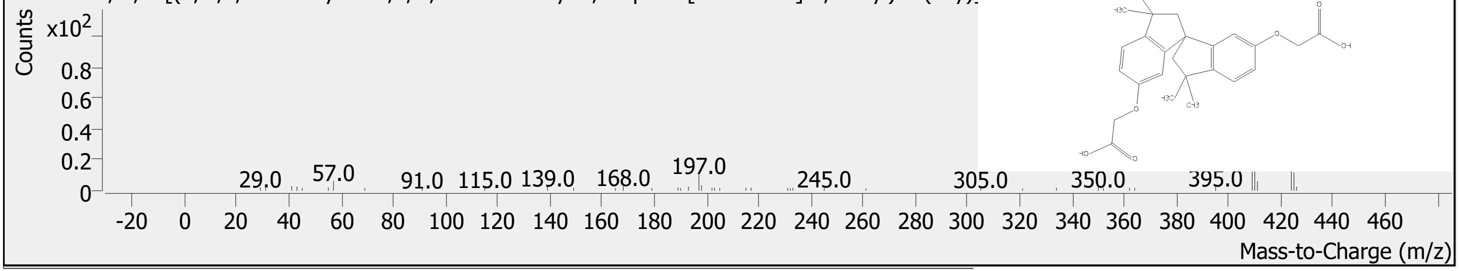

RT	Compound Name	CAS#	Formula	Area	Match Score	Sample	Sample
14.0493	Cinnamic acid, .alpha.-[N-benzoylamino]-3,5-di-t-butyl-4-hydroxy-	95820-15-8	C25H31NO4	4234649045	44.1	20.52	100.00

Cinnamic acid, .alpha.-[N-benzoylamino]-3,5-di-t-butyl-4-hydroxy- (NIST17.L)

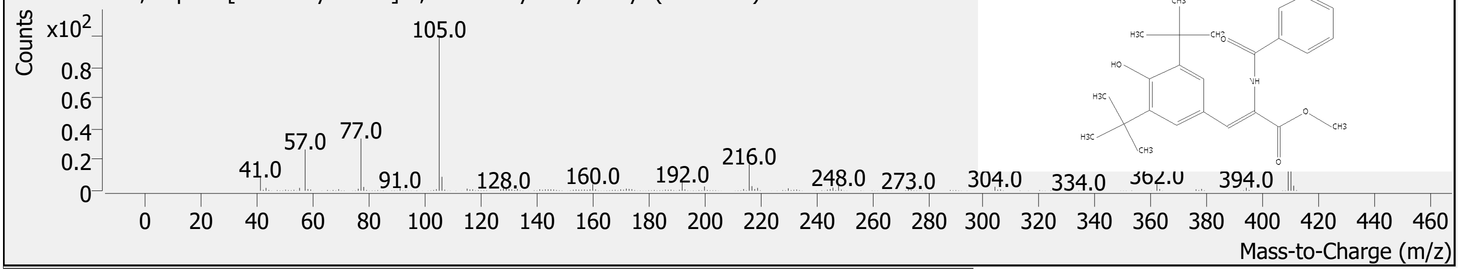

RT	Compound Name	CAS#	Formula	Area	Match Score	Sample	Sample
14.7580	Dioxybenzone, bis(tert-butyldimethylsilyl)-	1000453-70-1	C26H40O4Si2	3528779615	52.4	17.10	83.33

Dioxybenzone, bis(tert-butyldimethylsilyl)- (NIST17.L)

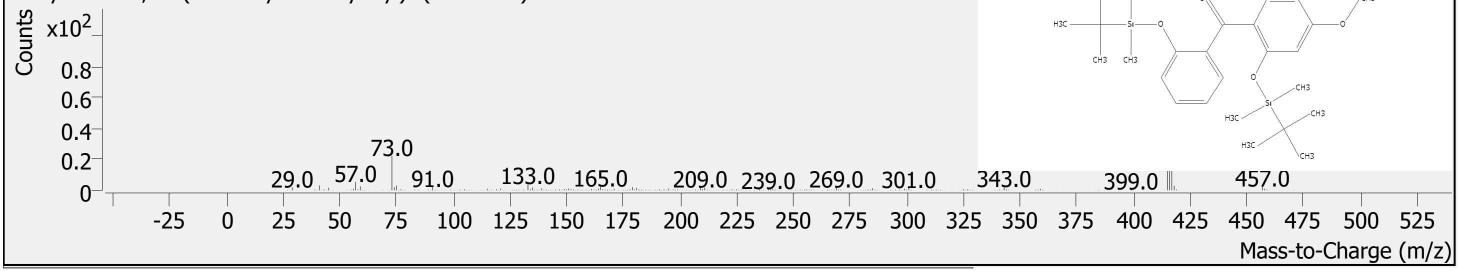

RT	Compound Name	CAS#	Formula	Area	Match Score	Sample	Sample
14.7580	Silane, diethyl(2-ethoxyethyloxy)octadecyloxy-	1000363-52-8	C26H56O3Si	3528779615	49.5	17.10	83.33

Silane, diethyl(2-ethoxyethyloxy)octadecyloxy- (NIST17.L)

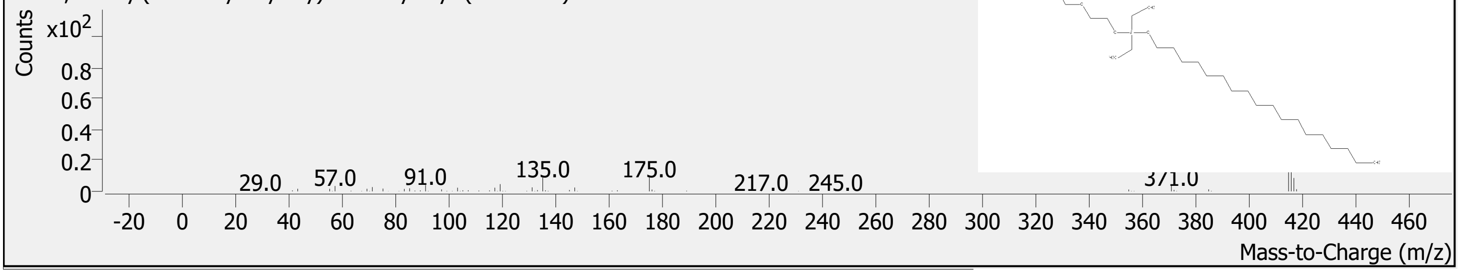

RT	Compound Name	CAS#	Formula	Area	Match Score	Sample	Sample
14.7580	3-(Tert.-butyldimethylsilyl)oxy-7,8,2',3'-tetramethoxyflavone	1000454-18-2	C25H32O7Si	3528779615	48.1	17.10	83.33

3-(Tert.-butyldimethylsilyl)oxy-7,8,2',3'-tetramethoxyflavone (NIST17.L)

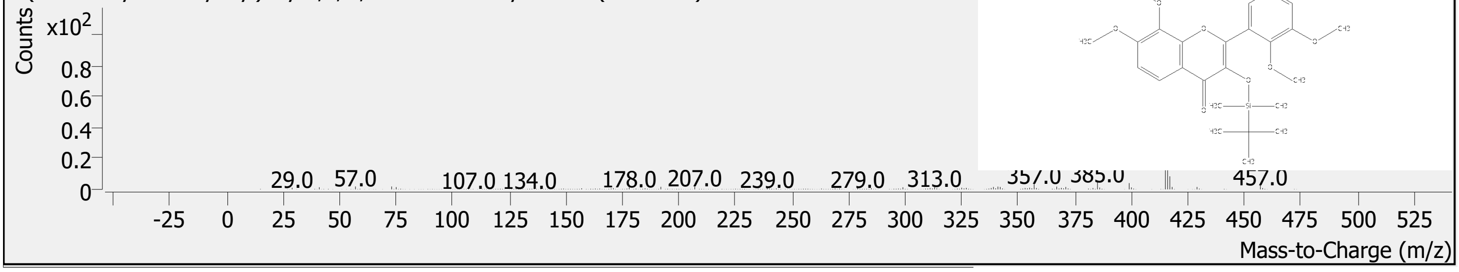

RT	Compound Name	CAS#	Formula	Area	Match Score	Sample	Sample
16.2165	8-(4-Methoxyphenyl)-11,11-dimethyl-8,10,11,12-tetrahydrobenzo[a][4,7]phenanthrolin-9(7H)-one	1000263-38-0	C25H24N2O2	128266688	39.0	0.62	3.03

8-(4-Methoxyphenyl)-11,11-dimethyl-8,10,11,12-tetrahydrobenzo[a][4,7]phenanthrolin-9(7H)-one (N

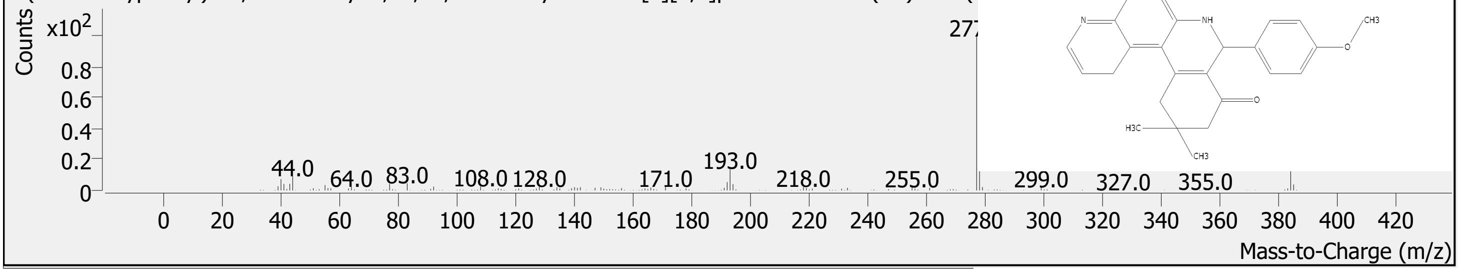

RT	Compound Name	CAS#	Formula	Area	Match Score	Sample	Sample
16.2165	Dimethoxymethyl-hydroxy-triphenyl phosphide	1000132-00-1	C21H23O3P	128266688	36.3	0.62	3.03

Dimethoxymethyl-hydroxy-triphenyl phosphide (NIST17.L)

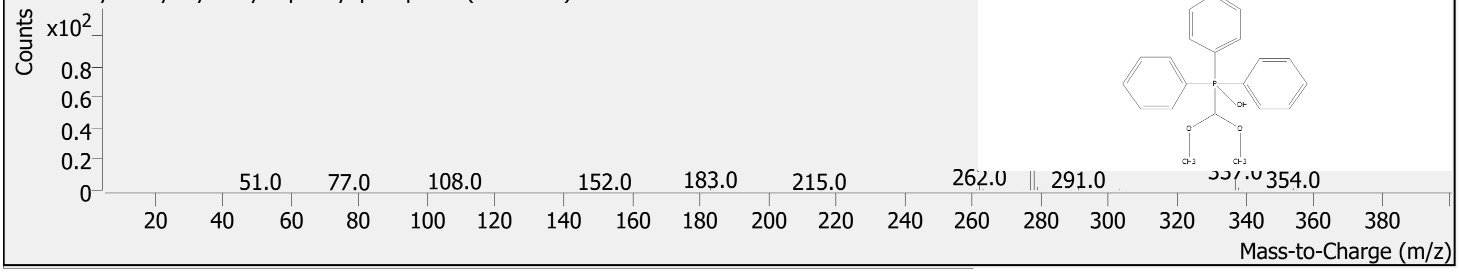

RT	Compound Name	CAS#	Formula	Area	Match Score	Sample	Sample
16.2165	Dibenzo[c,f]1,7-naphthyridin-9(7H)-one, 8,10,11,12-tetrahydro-11,11-dimethyl-8-phenyl-	1000271-48-2	C24H22N2O	128266688	34.9	0.62	3.03

Dibenzo[c,f]1,7-naphthyridin-9(7H)-one, 8,10,11,12-tetrahydro-11,11-dimethyl-8-phenyl- (NIST17.L

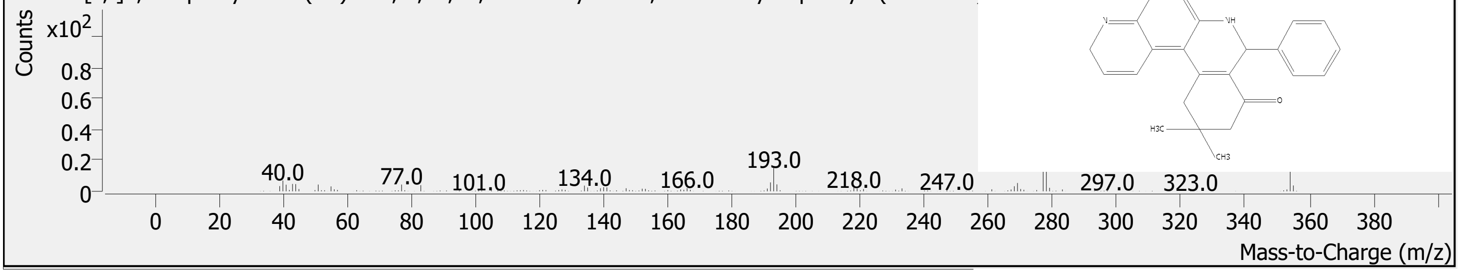

RT	Compound Name	CAS#	Formula	Area	Match Score	Sample	Sample
16.5461	4-(4-Acetylamino)-2,6-diphenylpyrimidine	130090-19-6	C24H19N3O	37095007	42.7	0.18	0.88

4-(4-Acetylamino)-2,6-diphenylpyrimidine (NIST17.L)

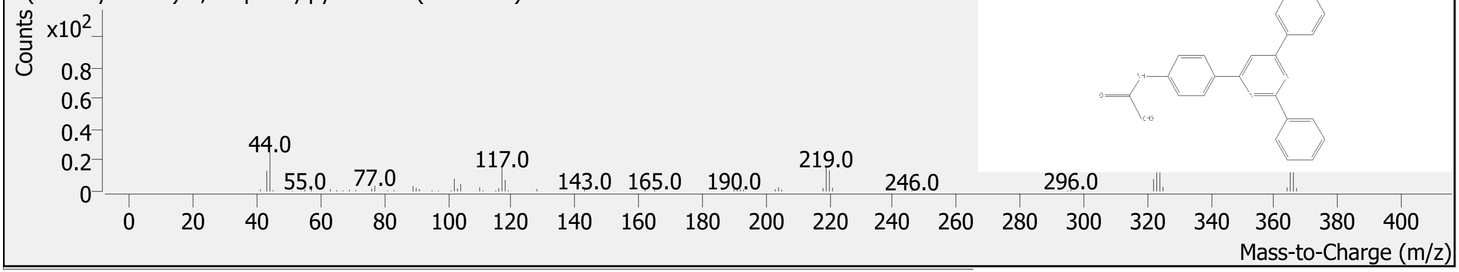

RT	Compound Name	CAS#	Formula	Area	Match Score	Sample	Sample
16.5461	2,5-piperazinedione, 3,6-bis[[4-(acetyloxy)phenyl]methylene]-	1000402-74-5	C22H18N2O6	37095007	41.9	0.18	0.88

2,5-piperazinedione, 3,6-bis[[4-(acetyloxy)phenyl]methylene]- (NIST17.L)

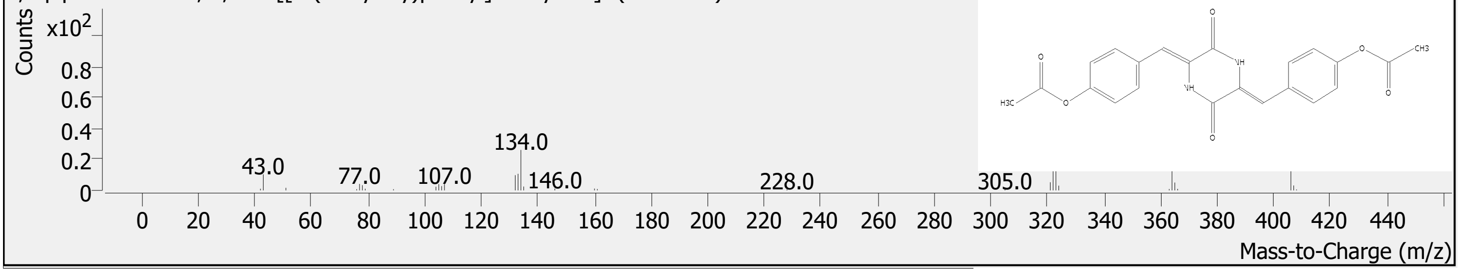

RT	Compound Name	CAS#	Formula	Area	Match Score	Sample	Sample
16.5461	1-Methyl-2,6-diphenyl-4,4-pentamethylene-1,4-dihydropyridine-3,5-dicarbonitrile	83078-31-3	C25H23N3	37095007	39.0	0.18	0.88

1-Methyl-2,6-diphenyl-4,4-pentamethylene-1,4-dihydropyridine-3,5-dicarbonitrile (NIST17.L)

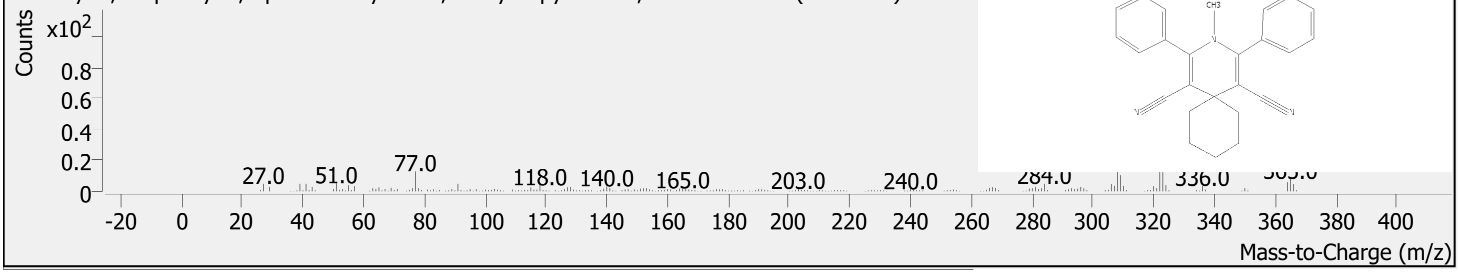

RT	Compound Name	CAS#	Formula	Area	Match Score	Sample	Sample
16.6614	Cyclodisilazane, 2,2,4,4-tetramethyl-1,3-bis(methyldiphenylsilyl)-	21116-67-6	C30H38N2Si4	91307132	34.6	0.44	2.16

Cyclodisilazane, 2,2,4,4-tetramethyl-1,3-bis(methyldiphenylsilyl)- (NIST17.L)

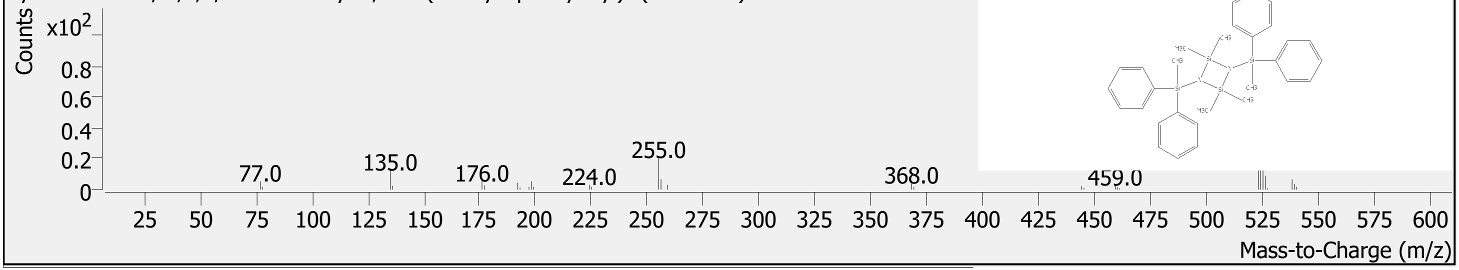

RT	Compound Name	CAS#	Formula	Area	Match Score	Sample	Sample
16.6614	6-Acetyl-1,3,8-trimethyl-7-phenyl-1H-imidazo[2,1-f]-purine-2,2(3H,8H)-dione	72793-12-5	C18H17N5O3	91307132	31.4	0.44	2.16

6-Acetyl-1,3,8-trimethyl-7-phenyl-1H-imidazo[2,1-f]-purine-2,2(3H,8H)-dione (NIST17.L)

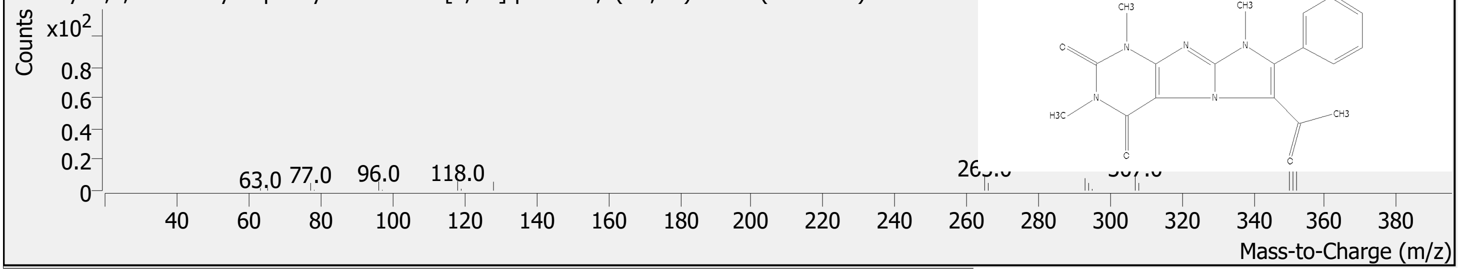

RT	Compound Name	CAS#	Formula	Area	Match Score	Sample	Sample
16.6614	.alpha.-Pyridone, 3,5-dimethyl-1,4,6-triphenyl-	344250-05-1	C25H21NO	91307132	29.9	0.44	2.16

.alpha.-Pyridone, 3,5-dimethyl-1,4,6-triphenyl- (NIST17.L)

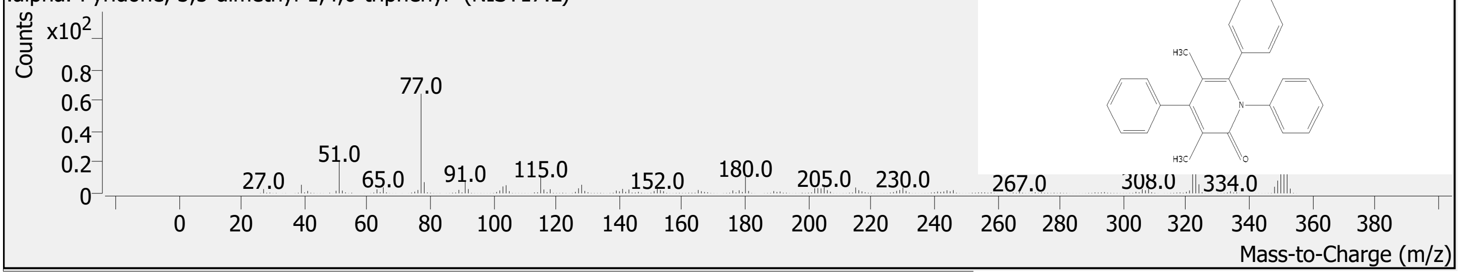

 Generated at 3:40 PM on 12/11/2020
