# Supplementary figures and images for: Broussonetia papyrifera Pollen Metabolome Insights, Allergenicity, and Dispersal in Response to Climate Change Variables
Source: Metabolites. 2025 Feb 18;15(2):137. doi: 10.3390/metabo15020137 (PMC11857163; doi:10.3390/metabo15020137)

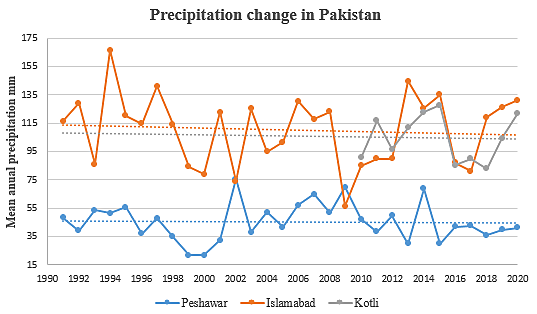

Supplement: Supplementary file 1 [file metabolites-15-00137-s001.zip › S1 Fig..tiff]

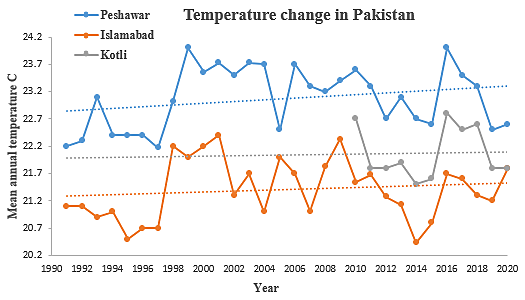

Supplement: Supplementary file 1 [file metabolites-15-00137-s001.zip › S2 Fig..tiff]

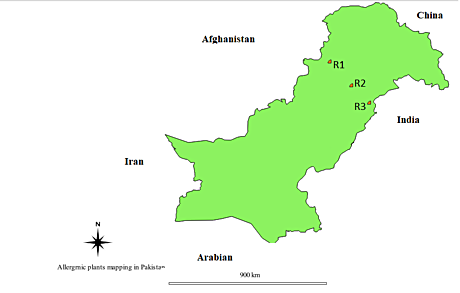

Supplement: Supplementary file 1 [file metabolites-15-00137-s001.zip › S3 Fig..tiff]

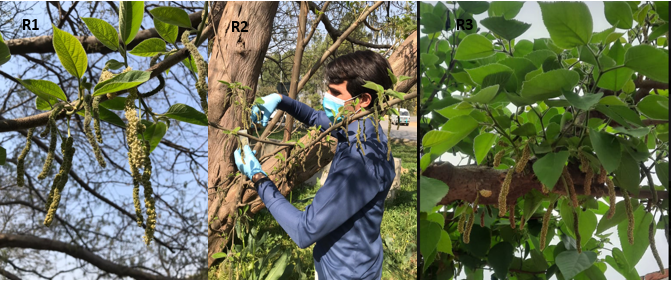

Supplement: Supplementary file 1 [file metabolites-15-00137-s001.zip › S4 Fig..tiff]

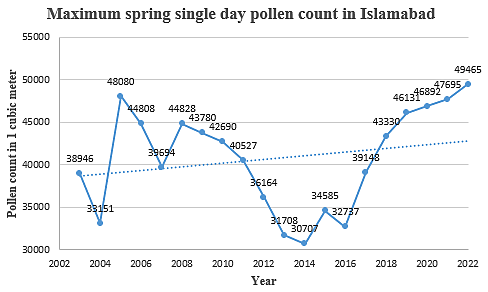

Supplement: Supplementary file 1 [file metabolites-15-00137-s001.zip › S5 Fig..tiff]
